# Stakeholders’ perspectives on the acceptability and feasibility of maternity waiting homes: a qualitative synthesis

**DOI:** 10.1186/s12978-023-01615-x

**Published:** 2023-07-05

**Authors:** Eva Julia van Braam, Daphne N. McRae, Anayda G. Portela, Jelle Stekelenburg, Loveday Penn-Kekana

**Affiliations:** 1 Faculty of Medicine, University of Groningen, Groningen, The Netherlands; 2 Department of Community Health and Epidemiology, University of Saskatchewan, Saskatoon, SK Canada; 3 Department of Maternal, Newborn, Child, and Adolescent Health and Ageing, World Health Organization, Geneva, Switzerland; 4 Department of Health Sciences, Global Health Unit, University Medical Centre Groningen/University of Groningen, Groningen, The Netherlands; 5 Department Obstetrics and Gynaecology, Leeuwarden Medical Centre, Leeuwarden, The Netherlands; 6 Department of Maternal Health and Health Systems, London School of Hygiene and Tropical Medicine, London, UK

**Keywords:** Maternity waiting homes, Safe motherhood, Maternal and newborn health services, Care-seeking

## Abstract

**Background:**

Maternity waiting homes (MHWs) are recommended to help bridge the geographical gap to accessing maternity services. This study aimed to provide an analysis of stakeholders’ perspectives (women, families, communities and health workers) on the acceptability and feasibility of MWHs.

**Methods:**

A qualitative evidence synthesis was conducted. Studies that were published between January 1990 and July 2020, containing qualitative data on the perspectives of the stakeholder groups were included. A combination of inductive and deductive coding and thematic synthesis was used to capture the main perspectives in a thematic framework.

**Results:**

Out of 4,532 papers that were found in the initial search, a total of 38 studies were included for the thematic analysis. Six themes emerged: (1) individual factors, such as perceived benefits, awareness and knowledge of the MWH; (2) interpersonal factors and domestic responsibilities, such as household and childcare responsibilities, decision-making processes and social support; (3) MWH characteristics, such as basic services and food provision, state of MWH infrastructure; (4) financial and geographical accessibility, such as transport availability, costs for MWH attendance and loss of income opportunity; (5) perceived quality of care in the MWH and the adjacent health facility, including regular check-ups by health workers and respectful care; and (6) Organization and advocacy, for example funding, community engagement, governmental involvement. The decision-making process of women and their families for using an MWH involves balancing out the gains and losses, associated with all six themes.

**Conclusion:**

This systematic synthesis of qualitative literature provides in-depth insights of interrelating factors that influence acceptability and feasibility of MWHs according to different stakeholders. The findings highlight the potential of MWHs as important links in the maternal and neonatal health (MNH) care delivery system. The complexity and scope of these determinants of utilization underlines the need for MWH implementation strategy to be guided by context. Better documentation of MWH implementation, is needed to understand which type of MWH is most effective in which setting, and to ensure that those who most need the MWH will use it and receive quality services. These results can be of interest for stakeholders, implementers of health interventions, and governmental parties that are responsible for MNH policy development to implement acceptable and feasible MWHs that provide the greatest benefits for its users.

*Trial registration* Systematic review registration number: PROSPERO 2020, CRD42020192219.

**Supplementary Information:**

The online version contains supplementary material available at 10.1186/s12978-023-01615-x.

## Abstract (highlights)

Maternity waiting homes (MWHs) are physical structures located close to a health facility to accommodate pregnant women before giving birth, providing easier access to maternal and newborn care services. Over the last decades MWHs have been implemented in countries worldwide as a strategy to increase facility-based birth with skilled health personnel, although global data on number of MWHs is not currently available. Large variation how MWHs are run, utilization and satisfaction rates have been observed in different contexts. We conducted a qualitative evidence synthesis to seek an in-depth understanding of this variability, exploring the perspectives of women, families, communities and health workers on the acceptability and feasibility of MWHs.

The decision-making process of women and their families before seeking care at an MWH was found in the review to involve weighing the gains and losses that come with MWH use. This study presents an overview of the gains and losses of MWHs that were reported as perceived by women, families, health workers and communities. The gains could include access to quality and culturally-appropriate care and life-saving interventions. These are weighed against the potential disadvantages of MWH use, such as costs, food insecurity and domestic responsibilities. Additionally, active community involvement and a sense of ownership were identified as key elements by community members for creating an acceptable and feasible MWH. In conclusion, successful implementation of MWHs is highly dependent on the context; engaging women, their families, health workers and the community in all phases of MWH implementation could increase acceptability and feasibility.

**Supplementary Information:**

The online version contains supplementary material available at 10.1186/s12978-023-01615-x.

## Background

Between 1990 and 2015, the global maternal mortality ratio has dropped by nearly 45% [1]. Despite this progress, maternal mortality remains unacceptably high with an estimated 295,000 maternal deaths and 2.6 million stillbirths worldwide per year. [2] Low- and middle-income countries (LMICs) account for approximately 94% of all maternal deaths [3]. Understandably, maternal and newborn health remains a high priority on the global health agenda and is identified as a key concern in the third Sustainable Development Goal [4].

Maternal deaths are predominantly the result of ‘direct’ causes from pregnancy and childbirth, such as obstructed labour, obstetric infection, haemorrhage, (pre)eclampsia and unsafe abortion [5]. Most of these complications are preventable or treatable when timely access to adequate healthcare is available. The World Health Organization (WHO) estimates that globally 81% of births were assisted by skilled health professionals between 2014 to 2019, ranging from 61% in sub-Saharan Africa to 99% in Europe, Central Asia and North America [6].

Maternity waiting homes (MWHs) are an intervention recommended by the WHO to increase access to maternity care services and increase facility-based births [7, 8]. MWHs are defined as residential lodging near, or within, a health facility that accommodates pregnant women during their final weeks of pregnancy, bringing them closer to a skilled health professional when labour starts [9]. In 1996, WHO issued a report with recommendations on key elements for MWHs [10]; however, no official standard guideline for the implementation of MWHs have been published. Globally, MWHs fulfil varying roles in the maternity care chain and functioning varies in different settings. The main users of MWHs are women of reproductive age that have barriers to seek timely perinatal care, such as geographical or financial barriers, social restrictions and/or health illiteracy. Some MWHs provide extended services including antenatal care and postnatal care to the mother and newborn, as well as health education, including information on care of the woman, care of the newborn, and family planning. In addition, some MWHs include income-generation and/or skills development activities for women, such as gardening, sewing and finances [11–13].

Over the past decades, MWHs have been implemented in more than 25 countries worldwide [10]. In Cuba, Peru, Liberia and Ethiopia, the National Ministry of Health incorporated a nation-wide scale-up of MWHs in their national strategy to decrease maternal and neonatal mortality [14–17]. Previously, several qualitative studies focused on understanding the successful implementation of MWHs, including a qualitative review of 29 studies [18]. The uptake and acceptability of MWHs vary substantially, from fully acceptation in the community to under-utilization and dissatisfaction with MWHs [11, 19–25]. These findings raise questions about the feasibility of implementing a sustainable MWH [12, 22, 26]. It is common practice in evidence-to-decision frameworks to consider effectiveness as determined by quantitative studies and also to understand the acceptability and feasibility of an intervention by key stakeholders. Without the support of these stakeholders, including endorsement of the cultural acceptability of the MWH, the MWH is less likely to be used by the local community. This QES seeks to understand stakeholders’ perspectives (women, family, community and health workers), through answering the following research questions:. (1) What are women’s, families’, communities’ and health workers’ perspectives on the acceptability and feasibility of MWHs? (2) How are women’s, families’, communities’ and health workers’ perspectives of MWHs influenced by the MWH’s characteristics? (3) How are women’s, families’, communities’ and health workers’ perspectives of MWHs influenced by socio-economic status of women?

## Methods

Qualitative evidence synthesis is an umbrella term for research that is linked with systematically reviewing qualitative studies [27]. It is used to explore complex interventions and establish a great understanding of these interventions, therefore we selected this method to analyze qualitative evidence on stakeholders’ perspectives of MWHs [28]. Three steps were followed in conducting the qualitative evidence synthesis: (1) exploration and selection of the studies, (2) data extraction and coding and quality assessment, and (3) thematic synthesis.

This review’s protocol was registered with the International Prospective Register of Systematic Reviews (PROSPERO) [37], registration number CRD42020192219.

### Search strategy

An initial search was conducted by a research team from the University of Saskatchewan for this review, a MWH systematic review [29], and a MWH realist synthesis. This database search was conducted between 2 and 4 December 2019, using English, Portuguese and Spanish search terms. A detailed search strategy and search terms are included as Additional file 1.

A search was conducted of all MWH documents with qualitative content (qualitative and mixed-methods design with qualitative content), in the published and unpublished literature, based on title and abstract, in 13 electronic databases.

### Study selection

The following inclusion criteria were used for the selection of studies: (1) primary, qualitative studies and mixed-method studies with qualitative data, (2) that included women’s, families’, communities’ and health workers’ perspectives on the utilization of MWHs in LMICs, and (3) studies using qualitative methods for data analysis. Studies published from 1 January 1990 until 20 July 2020 were eligible for inclusion.

The following exclusion criteria was applied: (1) studies conducted in high-income countries, (2) quantitative studies, and (3) studies that did not include a qualitative analysis. There were no restrictions on language and publication status. Studies were not excluded based on quality.

Eligible studies from the initial search strategy were imported into EPPI-reviewer 4 [30]. After duplicates were eliminated, the inclusion/exclusion screening tool was piloted on 20 studies. This was followed by full-text screening, conducted independently by two reviewers. Differences were discussed between reviewers and where inconsistency remained, discussions were held with a third reviewer. Once at least 80% consistency was reached, the remaining studies were assessed against the inclusion criteria by one reviewer. Another independent reviewer conducted blinded screening on a 20% sample of all full-text documents to ensure validity. After the pilot, coding comparison showed high inter-rater agreement, ranging from 85 to 92% on inclusion/exclusion criteria.

### Study population

The study’s target population consisted of the following four subgroups: (1) women of childbearing age that have used an MWH (MWH users) or could have used an MWH but have not (MWH non-users), (2) families, including any type of family member of an MWH user or non-user, (3) community members, and (4) health workers, which include all types of health providers, including health staff from the MWH, adjacent health facility and referral hospital, health extension workers (HEWs), community health workers (CHWs) and traditional birth attendants (TBAs).

### Data extraction and analysis

We extracted the following information from the included studies: study characteristics including design, country, year and methods of data collection; demographics of the participants (including socio-economic status, age, ethnicity, gender); the MWH context including type of MWH, details of the setting, services offered; and the perspectives of each of the population groups, etc.

Initially 10 papers were used to pilot the data-extraction tool independently. Remaining data collection and coding was executed using a pre-set data extraction tool. We discussed and adjusted the tool on an iterative basis with the research team.

A combination of inductive and deductive coding was done on included studies in acknowledgement that the researchers came to the analysis with a number of questions but also wanted to allow new themes to emerge. Analysis with an inductive approach was conducted to create a set of ‘descriptive themes’ which are closely linked to the original findings from the primary studies [31]. An a priori coding tool was designed based on the themes found in the initial 10 papers; the codes were defined and applied on the remaining papers. New descriptive themes were added and the thematic framework was modified on an iterative basis. The analytical themes were discussed with an external advisory group and revised where necessary. Further elaboration on how the thematic synthesis was conducted can be found in Additional file 2.

### Quality appraisal

We used the Critical Appraisal Skills Program (CASP) Qualitative Studies Checklist to assess the quality of the included primary studies [32]. The tool contains 10 questions to assess the quality of the primary studies. This appraisal was conducted independently by two reviewers followed by the comparison and discussion of our assessment. We included all the articles in the review. W reviewed articles which were assessed as low quality to see if they introduced any new concepts or codes which were not mentioned in any higher quality papers. No new codes, concepts or ideas were identified in papers assessed as low quality [33].

### Reflexivity statement

The members of the review team were a mixture of academics, physicians, global health experts and students with a biomedical, public health, sociological and/or anthropological background. Each team member held prior beliefs on the maternal and newborn healthcare system based on their individual experiences in this field and several of the authors had conducted previous reviews on MWHs. Since this paper is a secondary analysis of data from original research, it should be noted that the data could include the primary researchers’ subjective interpretation. Potential risk of bias was addressed by continuing reflexivity within the team and by including the advice of an external advisory group and two larger meetings were organised to discuss and contextualize QES findings, from which various LMIC actors participated (mainly health workers and academics) During the data extraction the research team actively searched for opposing perspectives in papers as well as well as perspectives that were different from our own opinion and assumptions. Outlying perspectives were further analysed.

## Results

### Study selection

**Fig. 1:**
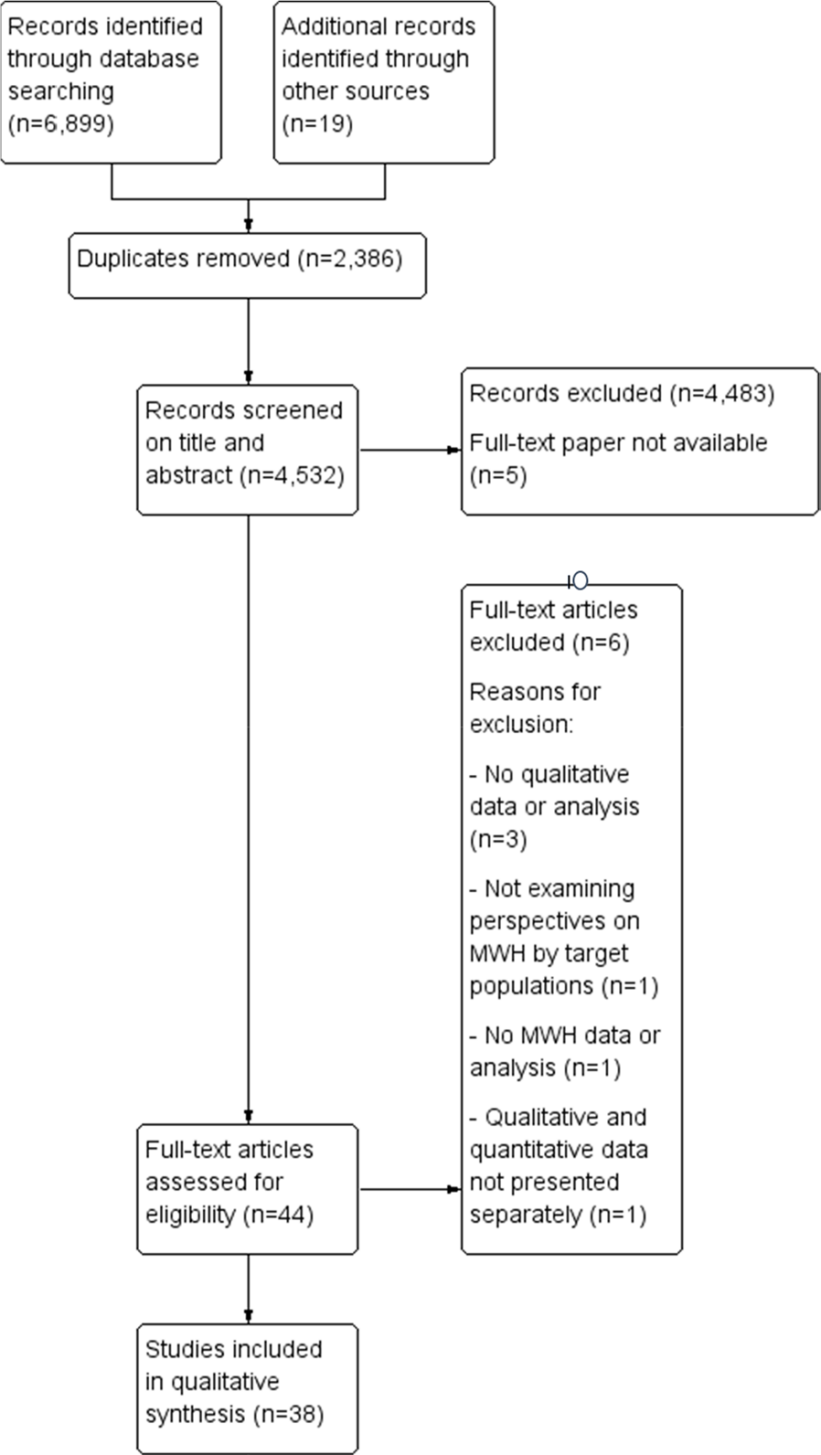
PRISMA flowchart for study eligibility screening

In total, 6899 records were identified by the initial database search. Through other sources, we identified 19 additional records. From the database, 2386 duplicates were removed, which left 4532 records for screening on eligibility based on title and abstract. Of the 4532 records, 4483 articles did not meet our inclusion criteria based on the title and abstract and were therefore excluded. We attempted to obtain full-text papers for the remaining 49 records, from which we were able to retrieve 44 records for the full-text screening. Six full-text papers were then excluded for not meeting our inclusion criteria; the reasons for exclusion are presented in the PRISMA flowchart (Fig. 1). This systematic assessment of eligible studies resulted in the inclusion of 38 eligible, full-text papers for the qualitative analysis.

### Study characteristics

Table 1 presents a summary of the characteristics of the included studies. Additional information is included in Additional file 3. Thirty-eight studies were included from seven LMICs: 29 studies were conducted in sub-Saharan Africa (Ethiopia [7], Ghana [1], Kenya [2]; Liberia [4], Malawi [3], Mozambique [2], Sierra Leone [1], Tanzania [1], Zambia [8]); six studies in Latin-America (Guatemala [2] and Nicaragua [4]); and three studies in Asia (Indonesia [2] and Nepal [1]). Most studies were conducted in rural settings, in some studies the context was not further specified.

**Table 1:** Summary of the characteristics of the included studies

First author, year of publication	Study location	Study setting	Study design and participants in qualitative methods
Abdulkadir, 2017	Kenya	One MWH	Mixed method: KIIs, 25 FGDs with non-users, women of childbearing age who had already given birth to a first child
Bergen, 2019	Ethiopia	24 MWHs	Qualitative study: 31 IDIs with HEWs
Bonawitz, 2019	Zambia	Two MWHs	Mixed-method study: Pre- and post-intervention comparison with four FGDs with MWH users
Chibuye, 2018	Zambia	17 facilities with MWH	Mixed-method study: 25 FGDs, 87 KIIs with MWH users, non-MWH users, SMAGs and neighbourhood health committees, district community medical officers/ nursing officers, health facility in-charges, senior women, partner agencies staff supporting RMNCH, women with spouse
Clensay, 2007	Nicaragua	One MWH	Qualitative study: 11 IDIs (MWH users, MWH staff, health workers, diplomat, FGD (MWH users), participant observations
Friedman, 2008	Nicaragua	One MWH	Qualitative study: IDIs (three MWH users, seven members of the staff team, three representatives of other non-profit organizations working in alliance with the MWH. Casual conversations with staff, pregnant women, participants in educational programmes)
Garcia Prado, 2012	Nicaragua	14 SILAIS	Mixed-method study: semi-structured surveys with KIIs and IDIs (12 MWH's support committee, 11 health workers, eight members of civil society organizations, eight local authorities, 10 community leaders
Gaym, 2012	Ethiopia	Three MWHs	Mixed-method study: FGDs (74 MWH users), site visits and documentation
Jarquín, 2015	Nicaragua	One MWH	Qualitative study: 38 semi-structured interviews (15 women users, 10 companions, 10 visitors of the women at homes), 4 FGDs (women and companions)
Kaiser, 2019	Zambia	10 rural health centres and 10 MWHs	Longitudinal qualitative study: 94 IDIs (nurses, midwives, non-skilled birth attendants, in-charge, district health officers)
Kebede, 2020	Ethiopia	Eight MWHs	Qualitative study: four FGDs (MWH users) and 18 IDIs (clinicians, HEWs, MWH non-users), observations of MWHs using checklists and field notes
Kyokan, 2016	Sierra Leone	10 MWHs	Qualitative study: two FGDs (non-users), IDIs (eight users, four non-users), KIIs (one HPA manager, four BWH hosts, one assistant community leader, one community health volunteer, one community health volunteer and village development committee), document review, assessment of MWHs
Lori, 2013a	Liberia	Four catchment areas with MWHs	Qualitative study: eight FGDs (MWH users, MWH non-users, family members of MWH users, or family members of non-MWH users) and 12 IDIs (10 clinic staff, one NGO staff, one Ministery of Health and Social Welfare staff)
Lori, 2013b	Liberia	Five health facilities with an MWH, five without an MWH	Mixed-method study: FGDs (46 traditional midwives) and logbook data collection
Lori, 2016	Zambia	Five health facilities with MWHs and 10 health facilities without MWHs	Qualitative study: IDI with semi-structured interview guide and 47 FGDs (46 community leaders and 500 SMAGs, husbands and women of childbearing age)
Lori, 2017	Liberia	Six MWHs: five receiving the newly built MWH intervention	Mixed-method study: secondary analysis of patient satisfaction and 60 semi-structured interviews (16 TBAs, five community midwives, 38 MWH users)
Lori, 2020	Liberia	119 MWHs (all MWHs in Liberia)	Mixed-method study: 113 IDIs (health providers), 115 FGDs (196 MWH users, 298 MWH non-users, 205 male partners, 82 chiefs, 163 community leaders, 221 TBAs), logbook reviews, Geographic Information System
Med solidar	Mozambique, Chiure	One MWH	Mixed-method study: semi-structured interviews, IDIs and FGDs (730 MWH users and non-users)
Mramba, 2010	Kenya	One MWH	Mixed-method study: 30 IDIs (MWH users)
Pujiharti, 2019	Indonesia	One MWH	Qualitative study: nine IDIs and FGDs (two MWH users, six health workers, two NGO members), observation study of relevant documents
Ruiz, 2013	Guatemala	Two MWHs	Qualitative study: 48 IDIs (18 MWH users, influential family members, four community leaders, five MWH administrative medical staff, seven comadronas, two medical staff from health centres, one district-level representative, six medical personnel from hospitals
Schooley, 2009	Guatemala	One MWH	Qualitative study: IDIs and three FGDs (21 MWH users and TBAs, 17 female advocates of the MWH, 12 male advocates, including spouses, NGO staff and community health workers), observations
Scott, 2018	Zambia	Four MWHs	Mixed-method study: 17 FGDs (33 women, 32 men, 38 TBA/SMAG, 32 mothers-in-law), 38 KIIs (16 health facility staff, nine CHWs, four traditional leaders, five community leaders, four community members), FL (59 women, 53 men and 55 elders)
Shresta, 2007	Nepal	Seven PHI: four sub-health posts, two health posts, and one PHCC	Qualitative study: 18 IDIs (MWH non-users) and 28 FDGs (communities, staff and chairpersons of management committee of health institutions)
Sialubanje, 2015	Zambia	One MWH and two health facilities without MWH	Qualitative study: 32 IDIs (six MWH users and 24 non-users)
Sialubanje, 2016	Zambia	Seven different health centers, villages, and families	Qualitative study: 24 IDIs (11 male partners of MWH users and 13 male partners of non-users)
Sitefane, 2013	Mozambique	Nine MWHs	Qualitative study: 32 FGDs (women in reproductive age, community leaders (men) and their counsellors)
Sri Hilmi, 2020	Indonesia	Two subdistricts with MWHs	Qualitative study: IDIs and FGDs (10 MWH non-users)
Sundu, 2017	Malawi	One hospital	Qualitative study: IDIs (15 MWH users)
Suwedi-Kapesa, 2018	Malawi	Three MWHs	Qualitative study: with six IDIs (health workers (three nurses, midwife, technicians), three guards and four FGDs (27 MWH users)
Tiruneh, 2016	Ethiopia	MWHs	Mixed-method study: 21 IDIs and surveys with open-ended questions (14 MWH users, six male partners of MWH users)
Urwin, 2017	Malawi	One MWH	Qualitative study: 6 IDIs and one FGD (six MWH users)
Van Rijn, 2013	Tanzania	One MWH	Mixed-method study: 25 semi-structured interviews (10 MWH users, eight MWH non-users, seven health workers)
Vermeiden, 2018	Ethiopia	One MWH	Mixed-methods study: FGDs (28 MWH users), seven IDIs (staff and users), document review
Vermeiden, 2019	Ethiopia	One MWH	Qualitative study: 33 IDIs and five FGDs (43 community members and 31 health workers)
Vian, 2017	Zambia	Four health facilities with MWHs and villages in each catchment area	Mixed-methods study: 16 FGDs (135 women who gave birth in the past 24 months, men with child under 24 months and community elders)
Wester, 2018	Ethiopia	Afar Regional Health Bureau	Qualitative study: 12 IDIs (health workers and gender experts with a formal university education)
Wilson, 1997	Ghana	One MWH	Qualitative study: 20 FGDs (57 community men, 52 community women, 14 trained TBAs, 24 hospital staff, eight Ghana Private Road Transport Union executives, eight relatives of women admitted with complications

*BWH* Birth Waiting Home, *CRHCs* Community Rural Health Centre, *KIIs* key-informant interviews, *DCMOs* District Community Medical Officers, *FGDs* focus group discussions, *IDIs* in-depth interviews, *NGO* non-governmental organization, *SILAIS* local systems of integrated health care at the regional level, *SMAGs* Safe Motherhood Action groups, *SNNP* Southern Nations Nationalities and People, *TBAs* traditional birth attendants

Most studies presented a mixture of the perspectives of the different population groups: 23 studies captured the perspectives of MWH users; 18 studies captured the perspectives of MWH non-users; 16 studies included the perspectives of health workers; seven studies presented the perspectives of families of users; and 18 studies included the perspectives of community members.

### Quality assessment

All but two studies included a clear statement of study aim. Qualitative methodology was appropriate for all included studies and most studies used an appropriate design to address the aim of the research, although not all studies justified their choice of methodology. Nearly all studies used an appropriate recruitment strategy. The majority of the studies conducted their research with ethical approval and obtained participants’ written consent, with the exception of four studies that did not report on ethics. Most of the studies did not elaborate on other ethical matters; however, risk of adverse effects in these type of studies is generally low. An ethical issue that could arise is regarding confidentiality; women may be afraid to express their opinions of quality for fear of consequences. Few studies reported on reflexivity and/or the relationship between the researcher and participants, with only three studies showing any form of reflexivity. Additionally, the included studies reported minimal considerations of divergent cases or views.

### Thematic analysis

#### Proposed framework acceptability and feasibility of MWHs’

**Fig. 2:**
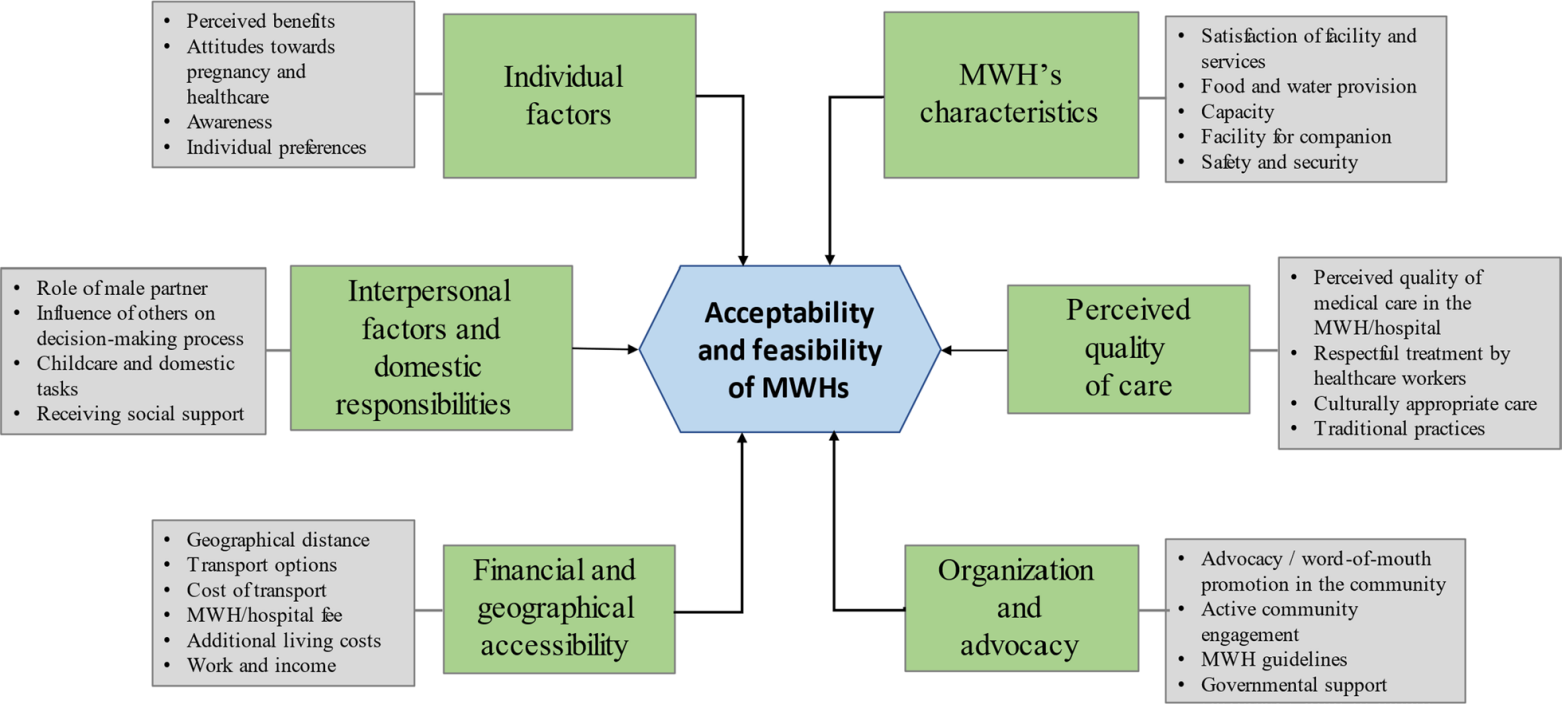
Thematic framework of the women’s, families’, communities’ and health workers’ perspectives on the acceptability and feasibility of MWHs

Our thematic synthesis resulted in six third-order, over-arching themes, which present the perspectives of the four population groups on the acceptability and feasibility of MWHs. The thematic analysis includes the synthesis of a hierarchical, tree-structure with the first-, second- and third-order themes (Additional file 4). The six third-order themes are captured in a thematic framework (Fig. 2). Differences in the qualitative approach, quality, setting and research methods of the initial papers prevented an accurate weight analysis. Therefore, all themes are presented as equally important and there is no specific order for their presentation.

### Theme 1: individual factors

#### Perceived benefits and individual preferences

Twelve studies reported that overall attitudes of MWH users, families, communities and health workers towards MWHs were positive [22, 34–44]. There were various perceived benefits from using MWHs, such as MWHs were recognized as a (life-saving) intervention that could contribute to better health outcomes of women and their newborns [7, 13, 19, 34, 35, 38, 39, 45–50]. MWH users perceived MWHs as a resting place, where women could take a break from their household and domestic tasks, which was perceived as beneficial before giving birth [13, 23, 38–41, 45, 48, 49]. All subgroups felt they had easier access to health services when using the MWH, including better monitoring by health staff and easier transfer to higher-level facilities when complications occurred [7, 12, 19, 24, 35, 37, 39, 41, 42, 50–53]. Other incentives for MWH users were health education [34, 49, 54], free lodging [13, 39], the possibility of avoiding negative experience with home births [37, 43], the benefits of sharing experiences and doing tasks together with other women [23, 38, 40, 41, 46, 50, 55] and the possibility of learning a new skill, such as sewing or gardening [13]. Two studies with health workers in adjacent facilities reported that it was easier to plan their work as women arrived to the health facility via the MWH and complications were recognized earlier which lowered the number of cases with complications [12, 56].

In contrast, in twelve studies some participants, mostly non-users, reported that they did not see the benefits of MWHs [24, 34, 43, 46, 48–50, 57–59]. For example, some women explained that they had a positive experience with home birth in the past or negative experiences with facility-birth or the healthcare system in general [22, 34, 36, 37, 43, 60]. In some cases, women expressed an individual preference for home-birth or they feared an operation if they used the MWH [22, 34, 36, 37, 43, 60].

#### Awareness of MWH

Awareness and recognition of MWHs were reported by numerous studies. Eight studies reported that there was generally high awareness about MWHs and their role [36, 37, 39, 50, 51, 59, 61, 62]. Three studies reported low awareness among women and communities regarding the existence of MWHs [34, 40, 49]. Uncertainty about how the MWH functions and when to go to the facility was reported mostly among non-users [34, 36, 37, 43, 48, 53].

### Theme 2: Interpersonal factors and domestic responsibilities

#### Decision-making process and social support

Overall, studies reported different levels of women’s decision-making power. Seven studies reported the male partner as the main decision-maker [24, 34, 37, 39, 49, 60, 63]. In some cases he would prohibit MWH use, which was reported by non-users as a main reason for not using a MWH [23, 34, 36, 39, 40, 47]. Common reasons for not supporting MWH attendance by family members included domestic responsibilities, no perceived benefits and costs [23, 34, 36, 39, 47]. In other cases, the male partner supported his wife in seeking care at the MWH [24, 36, 37, 43, 50]. With approval from the male partner, mother-in-law, other family members and friends, MWH use was less burdensome for women [24, 35–37, 43, 47, 50]. An additional facilitator was strong support from family and friends for MWH use, demonstrated through the provision of food and goods or accompaniment to the MWH [13, 24, 34, 36, 61]. Lack of support was a barrier for MWH use [13, 61].

#### Family and household commitment

In 16 studies, domestic responsibilities were reported as a key factor in the decision-making process. Non-users and their male partners reported this as a reason for MWHs non-use, especially when there was no one to take care of the children and household [24, 34–37, 42, 45, 47, 49, 50, 53, 55, 57, 59, 60, 63]. Two studies reported that the male partner refused or could not take care of the children [47, 54]. Contrarily, MWH use could be facilitated when others were resuming the domestic responsibilities in the mother’s absence [24, 34, 39, 43, 51]. Two studies reported fear of adultery during absence as a reason for MWH non-use [43, 63].

#### Community’s perspectives influence decision-making

Six studies reported that the community did not have a positive perception of the MWH for example because the facility did not meet the expectations of the community [7, 22, 34, 36, 49, 54]. Several studies reported that the communities had a negative perception of the women who used the MWH, because they were seen as lazy and/or as forfeiting a ‘natural’ or home-birth [22, 34, 36, 49, 54]. Two studies reported that the communities were positive about the MWHs [36, 39].

### Theme 3: Financial and geographic accessibility

User fees were generally not customary in most MWHs, the additional cost for birth in the adjacent health facility (such as hospital fee, cost for medical equipment or baby clothes, transportation costs), were perceived as unacceptably high by users and their partners [7, 13, 22, 24, 25, 34, 36, 43, 50, 59]. Women and partners felt that women could be refused admission when they were unable to meet these necessary requirements [7, 22, 24, 34]. MWH users, non-users and male partners indicated that the cost of staying in the MWH (cost for food) was a burden [24, 34, 36, 37, 39]. MWH users felt that their work and income would become negatively affected during their absence [34, 35, 39, 49, 59, 63]. Three studies reported that a fine had to be paid for home birth which was an incentive for women to use an MWH [7, 37, 61].

#### Geographical barriers and transport difficulties

Twelve studies reported transportation difficulties from their home to the MWH. MWH users, non-users and male partners acknowledged that arriving at the MWH was challenging when the MWH was not within walking-distance and transport options were scarce [7, 13, 22, 25, 34, 36, 37, 43, 49, 61, 64, 65]. Nine studies with users, non-users and their male partners and HCWs reported transportation challenges from the MWH to the adjacent health facility at the start of labour or in case of a complication [7, 19, 24, 34, 37, 42, 45, 59, 60]. Two studies reported that living in a rural area was a disadvantage in accessing MWHs, considering that MWH users’ households and families were unable to support women, specifically where facilities did not allow companions and the family had to travel long distances [7, 34, 50].

### Theme 4: MWH characteristics

#### Basic facilities and services

Satisfactory basic facilities facilitated MWH use and poor basic facilities lowered the satisfaction of MWHs [7, 24, 35, 38, 42, 49, 53, 60, 65]. Basic services that attracted women to MWHs were access to electricity, clean sanitary facilities, and cooking utensils [7, 22, 24, 34, 51]. MWH users frequently complained about lack of electricity [7, 19, 38, 42, 52], sanitary facilities [7, 22, 24, 34, 41, 42, 51], cooking facilities [34, 42, 49, 51, 65] and mosquito nets [23, 42, 65]. Thirteen studies with all four population groups reported poor state MWH conditions and low capacity, resulting in overcrowded facilities and a lack of privacy. Other studies reported women sleeping outside [7, 14, 19, 22, 24, 34, 41–43, 48–51]. Five studies noted that MWH users experienced boredom while staying at the MWHs and would have liked access to activities such as a television or income-generating activities [7, 42, 46, 49, 50]. Seven studies reported that women would like to have the possibility of bringing a companion to the MWH for mental and practical support [24, 25, 39, 40, 49, 51, 65]. Contrarily, some users and non-users noted that they were less likely use the MWH because they were allowed to bring a companions [13, 19, 42].

#### Food and drinking water insecurity

Food insecurity was highlighted in fifteen studies as a key barrier to MWH use according to all population groups, especially when there was no one to accompany the women during her stay in the MWH [7, 13, 14, 34, 36, 37, 39, 42, 46, 50, 55, 57, 61–63]. This was also the case for limited access to drinking water [7, 19, 22, 36, 38, 48, 52]. The provision of culturally-appropriate food and clean water made the MWH more attractive to women [7, 23, 34, 35, 37, 44, 48, 65].

### Theme 5: Perceived quality of care

#### Quality of care in the MWH/health facilities

It was difficult to analyse the quality of care between the MWH and other health facilities because most included papers did not report on the differences between quality of care in the MWH and health facility and/or the respondents in the studies did not distinguish between the quality of care in the MWH and the adjacent health facility because for example because they were both located in the same establishment or they were not asked to distinguish. A few studies that did compare the quality of care between the MWH and the health facility [24, 38, 39, 42, 47–49, 52, 63, 66]. In these papers, the regular check-ups by health workers was reported by stakeholders as important aspect of good medical care. Users, male partners and (community) health workers perceived the quality of care that was provided in the health facilities as satisfactory [7, 19, 24, 34, 35, 39, 40, 42, 45, 47, 49] or unsatisfactory [13, 22, 24, 25, 42, 45, 46, 50, 59, 63]. Disrespectful treatment by health workers in MWHs, including women being screamed at or criticized, was identified as one of the main reasons for unsatisfactory care and non-use in multiple studies [7, 23, 25, 36, 39, 41–43, 46, 53, 63]. On the contrary, many MWH users encountered respectful care by staff and a positive relationship and interaction with health workers [34–36, 43, 47, 49, 64].

Four studies highlighted that the perceived quality of care in the MWH, adjacent health facility or higher-level referral hospital was a key factor in the acceptability of MWHs. Women experienced poor quality of care in the adjacent or referral health facility [13, 41–43, 57, 60, 61, 65]. Only one study reported quality of care in the adjacent health facility as satisfactory [19].

Integration of cultural factors was highly valued by MWH users and family members, especially allowing traditional birth practices or assistance of a TBA [14, 36, 40, 47, 56, 60]. Care that was perceived as culturally inappropriate, such as provision of care by male health workers or the prohibition of traditional birth practices, often resulted in non-use [7, 34, 36, 40, 54, 63].

### Theme 6: Organization and advocacy

#### Advocacy and referral system

Five studies highlighted that women were affected in their decision-making process by the experiences and word-of-mouth promotion of others [34, 36, 46, 47, 50]. Health workers and community members suggested that TBAs, community leaders, community health workers, could effectively promote MWHs with the target population [6, 14, 28, 30, 32, 33, 44, 49, 53, 60]. HEWs helped to build community support for MWHs and these sensitization efforts improved acceptance of MWHs according to HEWs and health staff [9, 36, 40]. Other studies advised that health workers in the hospital and nurses or midwives in ANC clinics should be aware of MWHs so they can refer women to the facility [7, 43, 44, 47, 48, 57].

#### Community engagement

Health workers and community members highlighted active involvement of the community in different aspects of the MWH, to gain community support and make the MWH more acceptable to the community [23, 31, 32, 34, 36, 37, 39–42]. In addition, some studies with HEWs and health workers suggested that communities should contribute financially [23, 31, 33, 40].

#### Management and staffing issues

Notably, health workers frequently reported insufficient funding causing financial deficits as a key issue in the sustainability of MWHs [7, 13, 36, 40, 45, 49, 54]. They also highlighted the need for implementing standardized guidelines to facilitate processes within the MWH [13, 22, 49, 54].As the purpose of MWHs is to increase the number of women giving birth at health facilities, it is not surprising that the presence of a MWH resulted in health workers experiencing higher workload, in some instances [7, 22, 45]. The lack of governmental support and responsibility was highlighted by health workers in nine studies as the main cause of the management and financial challenges in MWHs [7, 22, 34, 36, 46, 47, 50, 54, 60].

## Discussion

The factors that influenced acceptability and feasibility among women, families, communities and health workers can largely be explained by the strategy of weighing the perceived gains and losses as described by Downe et al. [67]. Sufficient perceived benefits (gains) of MWH use should weigh up against the disadvantages (losses) that are associated with MWH use in order to make the MWH acceptable for women and their families.

An MWH is not a facility with one single function and can not be presented as a fixed model; it is a link in the maternal and newborn health system that can fulfil different roles, depending on the needs of the setting and the organization of the delivery of maternal and newborn health services in that area. Nonetheless, overarching aspects and concepts were identified that could provide valuable insights into the facilitators and barriers that make an MWH more acceptable and feasible according to women, families, health workers and communities, which are summarized in Additional file 5.

Our findings that are presented in Fig. 2 presents some essential components for consideration in regards to the acceptability and feasibility of MWHs. First of all, provision of decent accommodation with adequate living conditions is fundamental to the acceptability of MWHs which is consistent with the results of a previous literature review [18]. One of the key elements is the provision of food in the MWH, which had not previously been identified. The barrier of food insecurity is frequently related to the level of support women received from their social sphere, distance to MWH and financial situation.

Family commitments, work and loss of income during MWH attendance could prevent MWH use. These findings reflect the findings of a previous literature review on barriers and facilitators to MWH use [18]. This review also showed MWHs provided a place to rest which was perceived as beneficial during pregnancy.

Stakeholders frequently reported on access to respectful, culturally-appropriate, high quality care in the MWH and the affiliated health centre, which is consistent with previous literature on facility births [12, 68, 69]. Most included studies did not report separately on the perceived quality of care in the MWH and in the adjacent health facility, presumably because the MWH was often located inside or next to the health facility, and participants either could not, or were not asked to distinguish between the two. Subsequentially, the quality of care should be satisfactory in both the MWH and the health facility. A recent study advocated for the integration of culturally-sensitive and supportive maternity services [69]; this could be considered as a strategy for the maternal care in the MWH and the adjacent health facility. In this review although all articles shared the basic definition of a residential lodging near or within a health facility, the services provided in the MWHs, the level of facility that it was situated next to, how MWHs were financed and the population of women it aimed to serve varied. In many cases these aspects were not described in detail. Although a few of the papers described a model of MWHs as community-owned and run, financially sustainable and outside of the formal health system, many other articles discussed government-funded MWHs. Studies called for national government to take ownership and responsibility, to increase their involvement in funding and regulation, and to fully integrate MWHs within government service provision. Perspectives on acceptability and feasibility might differ between these community-owned and government-owned MWHs. Unfortunately, information on type of MWH was too limited in the initial papers to compare perspectives on the two types of MWH. Stakeholders did report on the absence of international MWH guidelines, including a monitoring and evaluation procedure [13, 18, 30, 32–34, 39, 42–44].

### Conceptual model

**Fig. 3:**
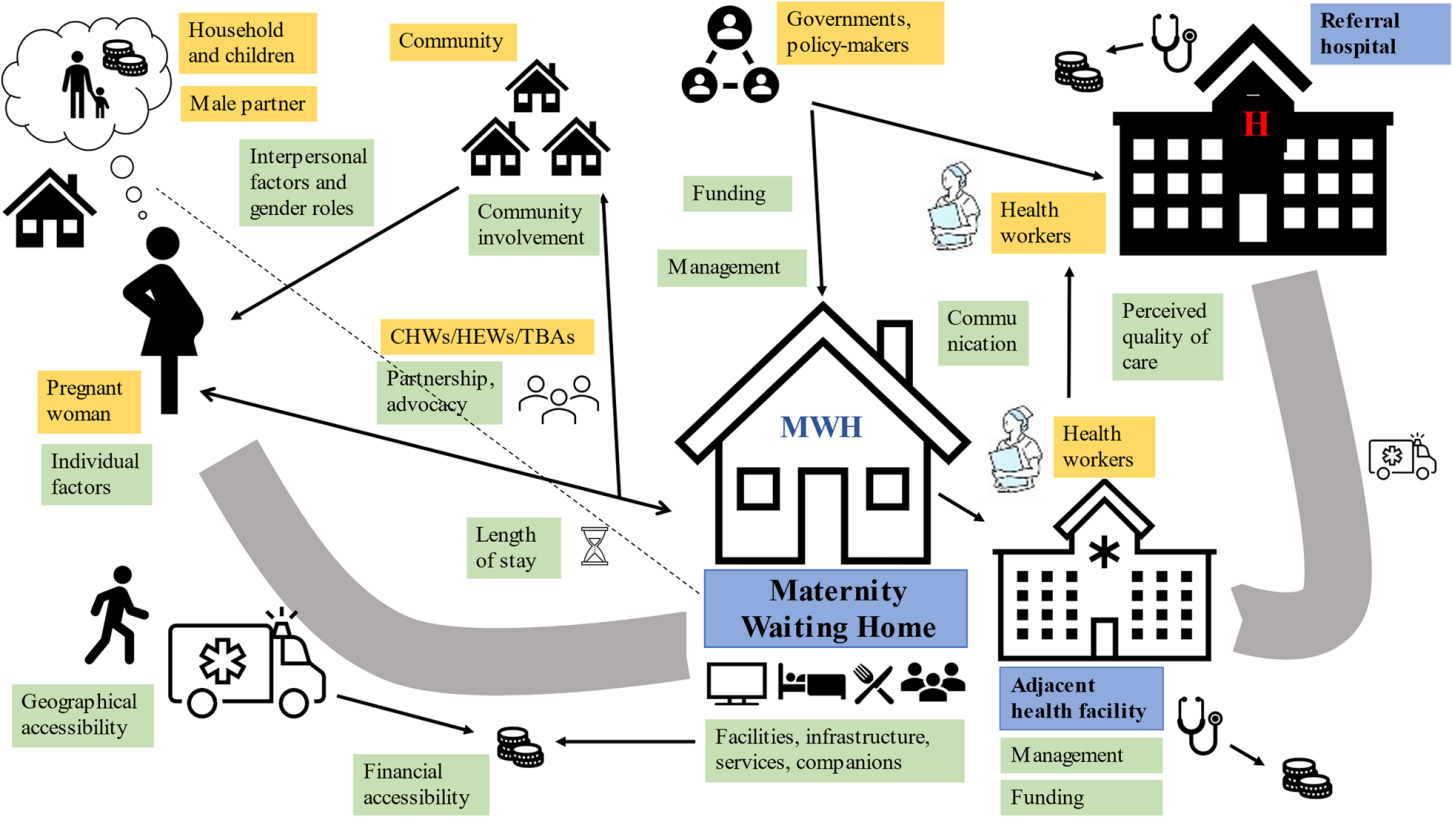
Conceptual model of determinants of MWH use. Yellow boxes: stakeholders; blue boxes: health facilities; green boxes: determinants of acceptability and feasibility of MWHs

A conceptual model was designed to illustrate the dynamic system of stakeholders (women, families, communities and health workers), MWH, adjacent health facility, referral hospital and the determinants of MWH use (Fig. 3) [70].

Few data was available on information about socio-economic background of the women and their families. Some studies found that women with a lower socio-economic status were the main users of MWHs [11, 71], other studies reported underutilization by women with a lower socio-economic status [34, 72, 73]. Our qualitative analysis revealed that socioeconomic factors, such as cost of living in the MWH, cost of transport to the MWH, hospital fees in the adjacent health facility, imposed significant barriers to MWH use. Most studies predominantly presented the views of women and their partners who were already attending MWHs and/or other maternal and newborn health services.

### Strengths and limitations

This is the first review with a robust systematic approach that evaluates the qualitative literature, including an extensive literature search and a quality assessment of studies.We have provided a rich overview of different factors that influence the acceptability and feasibility of MWHs as perceived by key stakeholders.

Nonetheless, there are several limitations with this review. For the synthesis of the results, we acknowledge that there may be a level of our own interpretation in the review that could not be prevented. By using a systematic approach and describing our rigorous analysis method in detail, we strived to increase the transparency of our findings. Part of our method of analysis was to include multiple reviewers in the coding and analysing process. We also reflected on our emerging conceptual themes with an external advisory team. Both methods were applied to reduce the risk of researcher bias.

Furthermore, we aimed to conduct inter-subgroup comparison of perspectives of users, non-users, families, health workers and communities. We aimed at differentiating between the views of MWH users and MWH non-users. Even though the cumulative numbers of the included MWH non-users in the primary studies were higher than MWH users, the presented perspective(s) of MWH non-users were often combined with the views of MWH users. Only four papers presented the perspectives of non-users separately [37, 45, 57, 66]. Therefore it was difficult to make a clear differentiation and comparison between these two groups of women. Exploring the views of non-users is essential for increased uptake of MWHs. If we had only focused on MWH users’ perspectives, the results would be less balanced—presumably representing more optimistic outcomes than reality.

Finally, the primary studies were heterogenous in study design, setting, sample size and participants, and the nature of the MWH programme they described varied and often lacked detail, which made it difficult to synthesise the findings. Details on study setting, study methodology and participants were provided in an attempt to preserve the context, but a certain level of de-contextualization could not be prevented. By providing this information, we hope that readers of the review are able to judge for themselves the extent in which the results are generalizable and transferable to other contexts.

#### Future research and implementations for practice

Community engagement has been identified in a large part of the literature as essential to ensure MWHs are used [23, 31, 32, 34, 36, 37, 39–42]. However what this engagement entails; how to make it effective; who should represent the community; how to ensure vulnerable groups are not excluded; and how community engagement works when women are travelling large distances, outside their community to MWHs, all requires further research.

We would have liked to conduct a sub analysis of between different characteristics that can make women vulnerable to not receiving medical care. However, we felt that this was beyond the scope of our review. These groups may experience different opinions about the intervention. An intersectional approach could be useful in future research to ensure the inclusivity of all groups that could benefit from MWHs. For example, The National Institute for Health and Care Research published a guidance for including under-served groups in social research [74], which could guide local researchers to identify under-served groups. There is a need to engage local researchers in setting the research-agenda, as this contributes to increased inclusiveness and helps bridge health inequities [75].

Financial factors played a great role in MWH use, both on patient-level, and organizational-level to maintain MWHs. Governmental support is fundamental to coordinate sustainable financial streams to ensure high quality care in MWHs, local and higher level medical facilities. This review exclusively focused on the perspectives of the selected stakeholders. Several stakeholders noted that MWHs encounter financial deficits and lack sufficient internal and external monetary sources. Including the views of local governments and other policy-makers in future research could help further explore the feasibility of MWHs in practice and the potential of making MWHs more financial sustainable [7, 13, 36, 47].

Implementation for practice is multifaceted. A main action point is toengage all parties in maternal healthcare in designing and evaluating the functioning of MWHs. Another recommendation is to conduct a local assessment of the needs and perspectives of the local communities on MWHs, including basic elements for MWHs, and barriers and facilitators, In particular, extra attention should go to understanding the needs of under-served groups. Finally, national governments should take responsibility to ensure funding and regulation of MWHs. This QES is part of a review package of three reviews, including a systematic review and meta-analysis [29] and realist synthesis [76]. The latter aimed to develop a theory regarding what resources work to support uptake and scale-up of MWHs.

## Conclusion

MWHs have been recommended by the WHO to help bridge the geographical gap to MNH services. This systematic synthesis of the qualitative literature provides an update of the existing literature on factors that influence the acceptability and feasibility of MWHs to different stakeholders. Although it is important to include stakeholders’ perspectives when designing health services to ensure their quality and responsiveness, it appears this may not be common practice for designing MWHs. The stakeholders’ perspectives offer insight into what motivates use and satisfaction with MWH services. The complexity and scope of these determinants of utilization underlines the need for MWH implementation strategy to be guided by context. Building a strong referral system with good partnership between various key actors and involving local communities and key stakeholders in all phases of MWH implementation is fundamental in making MWHs successful. Future research is necessary to make MWHs more inclusive to all groups and therefore help bridge health disparities*.* We hope that this review encourages consideration of stakeholders’ voices by local policy makers and programme managers as they aim to improve MWH quality and uptake and increase access to MNH services. Additionally, this study provides implementers with the most current and comprehensive overview of the research, enabling them to make improvements to policies that guide the implementation of acceptable, user-centred and sustainable MWHs.

## Supplementary Information

**Additional file 1: Appendix S0**. Search strategy and search terms.

**Additional file 2: Appendix S1**. Thematic analysis.

**Additional file 3: Appendix S2**. Characteristics of the included studies.

**Additional file 4: Appendix S3**. Thematic framework with final themes.

**Additional file 5: Appendix S4**. Perceived gains/facilitators and perceived losses/barriers.

## Data Availability

All data generated or analysed during this study are included in this published article [and its Additional files].

## Abbreviations

ANC: Antenatal care
BWH: Birth waiting home
CASP: Critical appraisal skills program
CHW: Community health worker
CRHC: Community rural health centre
DCMO: District community medical officer
FGDs: Focus group discussions
FL: Free listing
GIS: Geographical information mapping
IDIs: In-depth interviews
LMIC: Low- and middle-income country
MWH: Maternity waiting home
NGO: Non-governmental organization
PNC: Postnatal care
QES: Qualitative evidence synthesis
QOC: Quality of care
RMNCH: Reproductive maternal, newborn and child health
SES: Socio-economic status
SILAIS: Sistema local de atención integral de la salud (local system for integrated health care
SMAGs: Safe motherhood action groups
SNNP: Southern Nations Nationalities and People
TBAs: Traditional birth attendants
TMs: Traditional midwives
WHO: World Health Organization
