# Supplementary material for: Stakeholders’ perspectives on the acceptability and feasibility of maternity waiting homes: a qualitative synthesis
Source: Reprod Health. 2023 Jul 5;20:101. doi: 10.1186/s12978-023-01615-x (PMC10324180; doi:10.1186/s12978-023-01615-x)
Supplement: Supplementary file 1 — Additional file 1: Appendix S0. Search strategy and search terms. [file 12978_2023_1615_MOESM1_ESM.pdf]

Additional file 1: Appendix S0. Search strategy and search terms

A search was conducted of all MWH documents with qualitative content (qualitative and mixed design with qualitative content, in the published and unpublished literature) between December 2 and 4, 2019 in 12 electronic databases (AMED, CINAHL, Cochrane Library-Pregnancy and Childbirth, EMBASE, Global Health, Google Scholar, OVID Medline, MASCOT/WOTRO, ProQuest Dissertations and Theses Global, PsychInfo, Pubmed, and Web of Science). In available databases, we requested weekly auto alerts for our search. Searches were re-run on 20 July 2020 in the databases that did not offer auto alerts. Boolean operators (AND and OR) were used to combine search terms. Examples of keywords use in the search include: wait* OR await* and facility* OR home* OR hut* OR shelter* OR area* OR ward* AND antenatal* OR prenatal* OR pregnan* OR mother* OR matern* OR birth* OR intrapartum OR labour OR labor OR childbirth OR childbearing OR "child bearing" OR delivery*. The terms were searched, adapted to meet each database's search requirements.

After our original search several English and foreign language titles using the terms "casa materna" or "hogares maternos" were noted, rather than MWH. As MWHs are widely used in South American and African countries, we explored whether using foreign language phrases for MWH in French, Portuguese, and Spanish could increase the number of studies identified. Running foreign language phases in Google Scholar, we were able to identify new Spanish and Portuguese language items. We then had one author and two coders who are native Spanish and Portuguese speakers identify frequent phrases for MWH in these languages and re-ran all database searches with the terms "casa(s) materna(s)", "hogar(es) materno(s)", "casa de parto", "casa da gestante", "casa de Gestante", "bebê e puérpera", "centro de parto humanizado", "centro de parto normal", "centro de parto natural". In addition, we added the LILACS (Latin American and Caribbean Health Sciences Literature) database to our search strategy.

In addition, reference lists of included items were manually screened and the 'grey' literature was examined for additional relevant records. Authors were solicited for MWH documents to retrieve further studies and book or dissertation chapters and unpublished records were searched.

To retrieve the full text of the papers we relied on online access. In addition to this, we used inter-library loan, contacted authors directly and consulted with libraries when the full text was not openly accessible. We tried access via multiple universities (University of Saskatchewan, London School of Hygiene and Tropical Medicine, University of Groningen) to access the full papers."

Boolean operators (AND and OR) were used to combine search terms. The following terms were searched, adapted to meet each database's search requirements

**English**
(((wait* OR await*) and (facilit* OR home* OR hut* OR shelter* OR area* OR ward*)) adj5 (antenatal* OR prenatal* OR pregnan* OR mother* OR matern* OR birth* OR intrapartum OR labour OR labor OR childbirth OR childbearing OR "child bearing" OR deliver*)).
**Spanish and Portuguese**
"casa(s) materna(s)", "hogar(es) materno(s)", "casa de parto", "casa da gestante", "casa de Gestante", "bebê e puérpera", "centro de parto humanizado", "centro de parto normal", "centro de parto natural".
