# Supplementary material for: Stakeholders’ perspectives on the acceptability and feasibility of maternity waiting homes: a qualitative synthesis
Source: Reprod Health. 2023 Jul 5;20:101. doi: 10.1186/s12978-023-01615-x (PMC10324180; doi:10.1186/s12978-023-01615-x)
Supplement: Supplementary file 2 — Additional file 2: Appendix S1. Thematic analysis. [file 12978_2023_1615_MOESM2_ESM.pdf]

Additional file 2: Appendix S1. Thematic analysis

We used thematic analysis as the analysing method in the qualitative evidence synthesis, which is commonly used in qualitative systematic reviews (25). This methodology intends to collect primary qualitative studies on the subject, identify key issues in these primary studies and generate higher-perspective, 'analytical themes' (72). Codes were structured in hierarchical structure or a so-called 'tree-structure' that consist of first-order, second-order and third-order themes. Looking at the similarities and differences between the codes, this hierarchical structure was generated by EJB and LPK integrating into a conceptual framework the key themes that represented by the perspectives of the four subgroups. This conceptual framework was scrutinised by all members of the research team for conceptual coherence. A wider advisory team, that consists of research and/or clinical experts in the field of MWHs, was consulted and asked for their technical inputs on the underlying concepts and themes.
