# Supplementary material for: Stakeholders’ perspectives on the acceptability and feasibility of maternity waiting homes: a qualitative synthesis
Source: Reprod Health. 2023 Jul 5;20:101. doi: 10.1186/s12978-023-01615-x (PMC10324180; doi:10.1186/s12978-023-01615-x)
Supplement: Supplementary file 3 — Additional file 3: Appendix S2. Characteristics of the included studies. [file 12978_2023_1615_MOESM3_ESM.pdf]

Additional file 3: Table S1. Study characteristics of the included studies.

First author, year of publication	Study Location	Study setting	Study design and participants in qualitative methods	Sampling method (SM) analysis method (AM) Thematic framework (TF)
**Abdulkadir, 2017**	Kenya	One MWH	Mixed method: KIIs, 25 FGDs with non-users, women of childbearing age who had already given birth to a first child.	**SM:** purposeful sampling<br>**AM**: thematic analysis<br>**TF**: The PRECEDE – PROCEED theoretical model postulated by Green and Kreuter (1999)
**Bergen, 2019**	Ethiopia	24 MWHs	Qualitative study: 31 IDIs with HEWs	**SM:** purposeful sampling<br>**AM**: thematic content analysis<br>**TF**: Ecological model of social determinants of maternal health
**Bonawitz, 2019**	Zambia	Two MWHs	Mixed-method study: Pre- and post-intervention comparison with four FGDs with MWH users	**SM:** women who had been staying longest in MWHs<br>**AM:** Content analysis with themes comparison over time<br>**TF:** -
**Chibuye, 2018**	Zambia	17 facilities with MWH	Mixed-method study: 25 FGDs, 87 KIIs with MWH users, non-MWH users, SMAGs and neighbourhood health committees, district community medical officers/ nursing officers, health facility in-charges, senior women, partner agencies staff supporting RMNCH, women with spouse	**SM:** purposeful sampling<br>**AM:** thematic analysis<br>**TF: -**
**Clensay, 2007**	Nicaragua	One MWH	Qualitative study: 11 IDIs (MWH users, MWH staff, health workers, diplomat, FGD (MWH users), participant observations	**SM**: -<br>**AM**: -<br>**TF:** -
**Friedman, 2008**	Nicaragua	One MWH	Qualitative study: IDIs (three MWH users, seven members of the staff team, three representatives of other non-profit organizations working in alliance with the MWH. Casual conversations with staff, pregnant women, participants in educational programmes)	**SM:** -<br>**AM:** -<br>**TF:** Maternal Mortality and Structural Violence
**Garcia Prado, 2012**	Nicaragua	14 SILAIS	Mixed-method study: semi-structured surveys with KIIs and IDIs (12 MWH's support committee, 11 health workers, eight members of civil society organizations, eight local authorities, 10 community leaders.	**SM**: purposeful sampling and convenience sampling<br>**AM:** -<br>**TF:** -
**Gaym, 2012**	Ethiopia	Three MWHs	Mixed-method study: FGDs (74 MWH users), site visits and documentation.	**SM:** convenience sampling<br>**AM:** thematic analysis<br>**TF:** -
**Jarquín, 2015**	Nicaragua	One MWH	Qualitative study: 38 semi-structured interviews (15 women users, 10 companions, 10 visitors of the women at homes), 4 FGDs (women and companions)	**SM:** convenience sampling<br>**AM:** inductive methods for data analysis<br>**TF:** Donabedian's framework

First author, year of publication	Study Location	Study setting	Study design and participants in qualitative methods	Sampling method (SM) analysis method (AM) Thematic framework (TF)
**Kaiser, 2019**	Zambia	10 rural health centres and 10 MWHs	Longitudinal qualitative study: 94 IDIs (nurses, midwives, non-skilled birth attendants, in-charge, district health officers).	**SM:** purposeful sampling based on convenience<br>**AM:** content analysis using a mixed inductive deductive approach<br>**TF:** World Health Organization's (WHO) Health System Framework
**Kebede, 2020**	Ethiopia	Eight MWHs	Qualitative study: four FGDs (MWH users) and 18 IDIs (clinicians, HEWs, MWH non-users), observations of MWHs using checklists and field notes	**SM:** purposeful sampling<br>**AM:** Thematic content analysis<br>**TF: '**A-frame' of access proposed by Thiede et al
**Kyokan, 2016**	Sierra Leone	10 MWHs	Qualitative study: two FGDs (non-users), IDIs (eight users, four non-users), KIIs (one HPA manager, four BWH hosts, one assistant community leader, one community health volunteer, one community health volunteer & village development committee), document review, assessment of MWHs	**SM:** FDG: purposeful sampling, KIIs: -<br>**AM:** framework analysis approach<br>**TF:** -
**Lori, 2013a**	Liberia	Four catchment areas with MWHs	Qualitative study: eight FGDs (MWH users, MWH non-users, family members of MWH users, or family members of non-MWH users) and 12 IDIs (10 clinic staff, one NGO staff, one Ministry of Health and Social Welfare staff)	**SM:** recruited by clinic staff and community leaders using the following inclusion criteria: speak English or Kpelle and (b) willing to share their own experiences and understanding of MWHs.<br>**AM:** qualitative content analysis<br>**TF:** Penchansky and Thomas's (1981) predefined concepts of access
**Lori, 2013b**	Liberia	Five health facilities with an MWH, five without an MWH	Mixed-method study : FGDs (46 traditional midwives) and logbook data collection	**SM:** convenience sampling<br>**AM:** thematic analysis<br>**TF: -**
**Lori, 2016**	Zambia	Five health facilities with MWHs and 10 health facilities without MWHs	Qualitative study: IDI with semi-structured interview guide and 47 FGDs (46 community leaders and 500 SMAGs, husbands and women of childbearing age)	**SM:** convenience sampling<br>**AM:** latent content analysis and coding plan, Data were coded and grouped into 27 conceptual categories to facilitate abstraction. This process involved identifying units or segments of data and assigning a label or 'code word' that described the meaning and/or content.<br>**TF: -**

First author, year of publication	Study Location	Study setting	Study design and participants in qualitative methods	Sampling method (SM) analysis method (AM) Thematic framework (TF)
**Lori, 2017**	Liberia	Six MWHs: five receiving the newly built MWH intervention.	Mixed-method study: secondary analysis of patient satisfaction and 60 semi-structured interviews (16 TBAs, five community midwives, 38 MWH users)	**SM:** convenience sampling<br>**AM:** inductive content analysis to identify core themes related to patient satisfaction.<br>**TF: -**
**Lori, 2020**	Liberia	119 MWHs (all MWHs in Liberia)	Mixed-method study: 113 IDIs (health providers), 115 FGDs (196 MWH users, 298 MWH non-users, 205 male partners, 82 chiefs, 163 community leaders, 221 TBAs), logbook reviews, Geographic Information System	**SM:** interested participants from the community<br>**AM:** conceptual thematic analysis<br>**TF:** Glaser's constant comparative method
**Med solidar**	Mozambique	One MWH	Mixed-method study: semi-structured interviews, IDIs and FGDs (730 MWH users and non-users)	**SM:** random sampling<br>**AM: -**<br>**TF:** Legal framework on maternal and neonatal health
**Mramba, 2010**	Kenya	One MWH	Mixed-method study: 30 IDIs (MWH users)	**SM:** random sampling<br>**AM:** -<br>**TF:** -
**Pujiharti, 2019**	Indonesia	One MWH	Qualitative study: nine IDIs and FGDs (two MWH users, six health workers, two NGO members), observation study of relevant documents	**SM: -**<br>**AM:** interactive data analysis<br>**TF:** AGIL perspective by Ritzer
**Ruiz, 2013**	Guatemala	Two MWHs	Qualitative study: 48 IDIs (18 MWH users, influential family members, four community leaders, five MWH administrative medical staff, seven comadronas, two medical staff from health centres, one district-level representative, six medical personnel from hospitals	**SM:** snowball and convenience sampling<br>**AM:** thematic analysis with grounded theory methodology (Glaser and Strauss)<br>**TF**: -
**Schooley, 2009**	Guatemala	One MWH	Qualitative study: IDIs and three FGDs (21 MWH users and TBAs, 17 female advocates of the MWH, 12 male advocates, including spouses, NGO staff and community health workers), observations	**SM:** convenience sample<br>**AM:** thematic analysis.<br>**TF: -**
**Scott, 2018**	Zambia	Four MWHs	Mixed-method study: 17 FGDs (33 women, 32 men, 38 TBA/SMAG, 32 mothers-in-law), 38 KIIs (16 health facility staff, nine CHWs, four traditional leaders, five community leaders, four community members), FL (59 women, 53 men and 55 elders)	**SM:** FL: random sampling, purposeful sampling, KIIs sampling participants by recommending of FL respondents<br>**AM:** thematic framework analysis<br>**TF: '**Three Delays model' and the sustainability framework
**Shresta, 2007**	Nepal	Seven PHI: four sub-health posts,	Qualitative study: 18 IDIs (MWH non-users) and 28 FDGs (communities, staff and chairpersons of management committee of health institutions)	**SM:** selection of the site and participants for community FGDs by consulting the leader of the Village Development Committee

First author, year of publication	Study Location	Study setting	Study design and participants in qualitative methods	Sampling method (SM) analysis method (AM) Thematic framework (TF)
		two health posts, and one PHCC		**AM:** thematic analysis with pre-set themes and identification of emerging themes<br>**TF: -**
**Sialubanje, 2015**	Zambia	One MWH and two health facilities without MWH	Qualitative study: 32 IDIs (six MWH users and 24 non-users)	**SM:** purposeful sampling<br>**AM:** inductive approach by content analysis<br>**TF:** -
**Sialubanje, 2016**	Zambia	Seven different health centers, villages, and families.	Qualitative study: 24 IDIs (11 male partners of MWH users and 13 male partners of non-users)	**SM:** purposeful sampling in MWHs using multistage convenience sampling<br>**AM:** thematic content analysis<br>**TF:** -
**Sitefane, 2013**	Mozambique	Nine MWHs	Qualitative study: 32 FGDs (women in reproductive age, community leaders (men) and their counsellors)	**SM**: intentional sampling method<br>**AM:** thematic analysis<br>**TF:** -
**Sri Hilmi, 2020**	Indonesia	Two subdistricts with MWHs	Qualitative study: IDIs and FGDs (10 MWH non-users)	**SM:** -<br>**AM:** interactive technique of analysing data<br>**TF:** -
**Sundu, 2017**	Malawi	One hospital	Qualitative study: IDIs (15 MWH users)	**SM:** purposeful sampling<br>**AM:** thematic analysis<br>**TF:** -
**Suwedi-Kapesa, 2018**	Malawi	Three MWHs	Qualitative study: with six IDIs (health workers (three nurses, midwife, technicians), three guards and four FGDs (27 MWH users)	**SM**: convenience sample<br>**AM:** thematic analysis<br>**TF:** Donabedian and WHO maternity waiting homes conceptual frameworks
**Tiruneh, 2016**	Ethiopia	MWHs	Mixed-method study: 21 IDIs and surveys with open-ended questions (14 MWH users, six male partners of MWH users)	**SM:** convenience sample<br>**AM:** thematic framework analysis<br>**TF:** -
**Urwin, 2017**	Malawi	One MWH	Qualitative study: 6 IDIs and one FGD (six MWH users)	**SM**: random sampling of women at MWH<br>**AM:** -<br>**TF:** -

First author, year of publication	Study Location	Study setting	Study design and participants in qualitative methods	Sampling method (SM) analysis method (AM) Thematic framework (TF)
**Van Rijn, 2013**	Tanzania	One MWH	Mixed-method study: 25 semi-structured interviews (10 MWH users, eight MWH non-users, seven health workers)	**SM:** purposeful sampling<br>**AM:** thematic analysis with pre-set themes<br>**TF:** conceptual framework based on Thaddeus and Maine
**Vermeiden, 2018**	Ethiopia	One MWH	Mixed-methods study: FGDs (28 MWH users), seven IDIs (staff and users), document review.	**SM:** IDIs: purposeful sampling, FGDs: convenience sampling<br>**AM:** thematic framework analysis<br>**TF:** The Adapted Three Delay Model by Gabrysch and Campbell, WHO's four crucial elements of an MWH
**Vermeiden, 2019**	Ethiopia	One MWH	Qualitative study: 33 IDIs and five FGDs (43 community members and 31 health workers)	**SM:** purposive sampling, using the snowball technique.<br>**AM**: -<br>**TF:** Adapted Three Delays Model WHO Standards for Improving Quality of Maternal and Newborn Care in Health Facilities
**Vian, 2017**	Zambia	Four health facilities with MWHs and villages in each catchment area	Mixed-methods study: 16 FGDs (135 women who gave birth in the past 24 months, men with child under 24 months and community elders)	**SM:** multistage random sampling design, and purposive sampling<br>**AM:** thematic analysis with a priori themes.<br>**TF:** -
**Wester, 2018**	Ethiopia	Afar Regional Health Bureau	Qualitative study: 12 IDIs (health workers and gender experts with a formal university education)	**SM:** purposeful sampling<br>**AM:** thematic analysis<br>**TF:** -
**Wilson, 1997**	Ghana	One MWH	Qualitative study: 20 FGDs (57 community men, 52 community women, 14 trained TBAs, 24 hospital staff, eight Ghana Private Road Transport Union executives, eight relatives of women admitted with complications.	**SM:** -<br>**AM:** -<br>**TF:** -

Abbreviations: BWH: Birth Waiting Home, CRHCs: Community Rural Health Centre,KIIs: key-informant interviews, DCMOs: District Community Medical Officers, FGDs: focus group discussions, IDIs: in-depth interviews, NGO: non-governmental organization, SILAIS: local systems of integrated health care at the regional level, SMAGs: Safe Motherhood Action groups, SNNP: Southern Nations Nationalities and People, TBAs: traditional birth attendants, TMs: traditional midwives.
