# Supplementary material for: Stakeholders’ perspectives on the acceptability and feasibility of maternity waiting homes: a qualitative synthesis
Source: Reprod Health. 2023 Jul 5;20:101. doi: 10.1186/s12978-023-01615-x (PMC10324180; doi:10.1186/s12978-023-01615-x)
Supplement: Supplementary file 4 — Additional file 4: Appendix S3. Thematic framework with final themes. [file 12978_2023_1615_MOESM4_ESM.pdf]

Additional file 4: Appendix S2. Thematic synthesis

Third order theme: Individual factors

References	First order themes	Second order themes
(Bergen et al., 2019; Jarquín & Marenco, 2015; Kebede & Mihrete, 2020; Kyokan et al., 2016; Lori et al., 2017; Lori, Munro, et al., 2013; Ruiz et al., 2013; Scott et al., 2018; Susan Sundu & Ellen, 2017; Suwedi-Kapesa & Nyondo-Mipando, 2018; Vermeiden, 2019; Wester et al., 2018)	Positive perception on MWHs<br>*"In general, women reported positive experiences of using the BWHs: We liked the place. In fact, I was used to the place. I did not want to go back home even after I delivered. (User of BWH, female, 35 years, parity 3, all deliveries at PHU without adverse outcome, 2 days stay at BWH, Sella Limba chiefdom)" (Kyokan et al., 2016)*	Perceived benefits and risk perception
(Abdulkadir, 2017; Bergen et al., 2019; Chibuye et al., 2018; Clensay, 2007; Gaym et al., 2012; Jarquín & Marenco, 2015; Lori et al., 2017, 2020; Lori, Wadsworth, et al., 2013; Schooley et al., 2009; Sialubanje et al., 2015, 2016; Tiruneh et al., 2016; van Rijn, 2013; Vermeiden, Schiffer, et al., 2018)	MWH is contributing to better health care<br>*"Husbands of pregnant women admitted to MWHs believed in the advantages of staying at MWHs. They explained that woman would avert the risk of death due to late arrival in the health center (which is due to unavailability of an ambulance on time)." (Tiruneh et al., 2016)*	
(Gaym et al., 2012; Lori, Wadsworth, et al., 2013)	Benefit of free lodging	
(Abdulkadir, 2017; Gaym et al., 2012; Lori et al., 2017; Lori, Wadsworth, et al., 2013; Mramba et al., 2010a; Ruiz et al., 2013; Sialubanje et al., 2015; Susan Sundu & Ellen, 2017; Tiruneh et al., 2016)	Benefit of MWH as resting place<br>*"Women also reported they get a lot of rest despite their concerns over the problem of caring for their children/family at home." (Tiruneh et al., 2016)*	
(Clensay, 2007; Lori et al., 2017; Mramba et al., 2010a; Ruiz et al., 2013; Susan Sundu & Ellen, 2017; Tiruneh et al., 2016; van Rijn, 2013)	Benefit of sharing experiences and doing tasks together with other women	
(Lori et al., 2018; Lori, Munro, et al., 2013)	Benefit of easier to plan work by health workers	
(Clensay, 2007; Kaiser et al., 2019)	Benefits of MWH outweigh additional workload<br>*"Both health center staff and district health officers perceived that the benefits of the MWHs greatly outweighed the additional responsibilities previously discussed. (Kaiser et al., 2019)*	
(Bergen et al., 2019; Clensay, 2007; Pujihartati et al., 2020; Shrestha et al., 2007; Sialubanje et al., 2015, 2016; Tiruneh et al., 2016; van Rijn, 2013; Vermeiden, 2019; Wilson et al., 1997)	MWH use is unnecessary: low risk perception, home close to health services, no perceived benefits<br>*"Women from Adoagyiri, Pokuase and Dobro said they did not need the MWH because they were near enough to Nsawam Hospital." (Wilson et al., 1997)*	
(Bonawitz et al., 2019; Kebede & Mihrete, 2020; Kyokan et al., 2016; Lori, Wadsworth, et al., 2013; Sitefane et al., 2013; van Rijn, 2013; Vian et al., 2017; Wilson et al., 1997)	High awareness of MWHs<br>*"In general, there was a great deal of awareness about the MWH and its uses." (Wilson et al., 1997)*	Awareness and health literacy
(Bergen et al., 2019; Ruiz et al., 2013; Tiruneh et al., 2016)	Low awareness of MWHs<br>*"None of the women or family members, expect those who had been recruited from the service, had heard of MWHs." (Vermeiden et al., 2019)*	
(Bergen et al., 2019; Kebede & Mihrete, 2020; Kyokan et al., 2016; Sialubanje et al., 2015; Urwin, 2017; Vermeiden, 2019)	Uncertainty about when and how to use MWH, EDD unknown<br>*"Many women, both users and non-users of BWHs reported that they had heard about BWHs. However, they were uncertain about when to use the BWHs. While some women explained that women should go at the end of pregnancy, others reported that women should go there when labour has started." (Kyokan et al., 2016)*	
(Kyokan et al., 2016; Vermeiden, 2019)	Negative experience with home delivery	Individual preferences and experiences
(Bergen et al., 2019; Friedman, 2008a; Kebede & Mihrete, 2020; Kyokan et al., 2016; Scott et al., 2018; Vermeiden, 2019)	Preference for non-MWH birth: natural birth, fear of operation, home delivery, no perceived benefits	
(Friedman, 2008a; Kebede & Mihrete, 2020; Kyokan et al., 2016; Scott et al., 2018; Vermeiden, 2019)	Negative perception on health system in general	

Third order theme: Interpersonal factors and domestic responsibilities

References	First order themes	Second order themes
(Abdulkadir, 2017; Bergen et al., 2019; Friedman, 2008b; Jarquín & Marenco, 2015; Kebede & Mihrete, 2020; Kyokan et al., 2016; Pujihartati et al., 2020; Schooley et al., 2009; Sialubanje et al., 2016; Solidar Med, 2013; Suwedi-Kapesa & Nyondo-Mipando, 2018; Tiruneh et al., 2016; Urwin, 2017; van Rijn, 2013; Wilson et al., 1997)	No one to take care of the children and household, long time away from household<br>*"There are too many charlas (talks) and not enough medications. It is too far to travel and costs too much. Also who will take care of the children and cook and clean if my wife is away? (focus group participant)."* (Schooley et al., 2009)	Family/marriage commitments
(García Prado & Cortez, 2012; Schooley et al., 2009)	Male partner cannot to take care of household	
(Bergen et al., 2019; Bonawitz et al., 2019; Lori, Wadsworth, et al., 2013; Sialubanje et al., 2016; Vermeiden, 2019; Vermeiden, Braat, et al., 2018)	Others can take over the care for family and household in woman's absence<br>*"In addition to having a supportive husband, users were clear that support in the household was essential, which was mostly provided by family members."* (Vermeiden, Braat, et al., 2018)	
(Solidar Med, 2013; Vermeiden, 2019)	Fear of adultery during absence	
(Bergen et al., 2019; Friedman, 2008a; Kyokan et al., 2016; Lori, Wadsworth, et al., 2013; Sialubanje et al., 2016; Solidar Med, 2013; Tiruneh et al., 2016)	Male partner is the main decision-maker	Perspectives of others on MWH
(Bergen et al., 2019; Kebede & Mihrete, 2020; Lori, Wadsworth, et al., 2013; Mramba et al., 2010a; Ruiz et al., 2013; Schooley et al., 2009)	Male partner prevents MWH attendance	
(Kebede & Mihrete, 2020; Kyokan et al., 2016; Lori, Wadsworth, et al., 2013; Sialubanje et al., 2016; van Rijn, 2013; Vermeiden, 2019)	Male partner supports MWH attendance<br>*"The importance of men's roles was also acknowledged in the ability of a facility to accommodate pregnant women from the community: 'Yes, the community is very happy with the maternal waiting home, because the men in the community, when the women are here, they feel free that the women are secure.'"* (Lori, Wadsworth, et al., 2013)	
(Bergen et al., 2019; Mramba et al., 2010a; Ruiz et al., 2013; Vermeiden, 2019)	Mother-in-law is main decision-maker	
(Jarquín & Marenco, 2015; Schooley et al., 2009; Vermeiden, 2019)	Social network supports MWH attendance<br>*"Supportive friends, family members and advocates influenced the women´s decision to seek health care at the Casa Materna" (Schooley et al., 2007)*	
(Bergen et al., 2019; García Prado & Cortez, 2012; Kebede & Mihrete, 2020; Scott et al., 2018; Tiruneh et al., 2016)	Negative perspectives on women who use MWHs by the community	
(Chibuye et al., 2018; Kebede & Mihrete, 2020; Lori, Wadsworth, et al., 2013)	Positive perception on MWH by community	
(Bergen et al., 2019; Chibuye et al., 2018; García Prado & Cortez, 2012; Kebede & Mihrete, 2020; Scott et al., 2018; Tiruneh et al., 2019)	Negative perception on MWH by community	
(Bergen et al., 2019; Gaym et al., 2012; Kebede & Mihrete, 2020; Sialubanje et al., 2016; Sitefane et al., 2013)	Good support from family and relatives during MWH stay	Social support during MWH attendance
(Gaym et al., 2012; Sitefane et al., 2013)	Lack of support from family and relatives during MWH stay	

Third order theme: Perceived quality of care

References	First order themes	Second order themes
(Abdulkadir, 2017; Clensay, 2007; Lori et al., 2016; Scott et al., 2018; Sialubanje et al., 2016; Solidar Med, 2013; Suwedi-Kapesa & Nyondo-Mipando, 2018; van Rijn, 2013; Wilson et al., 1997)	Medical care in MWH perceived as unsatisfactory<br>*"The poor quality of healthcare services at the MWHs was another important barrier to MWH use. Husbands who believed that MWH healthcare staff did not conduct regular visits to check on the condition of pregnant women did not allow their wives to use the services."* (Sialubanje et al., 2016)	Perceived quality of medical care
(Friedman, 2008a; Gaym et al., 2012; Pujihartati et al., 2019; Shrestha et al., 2007; Sitefane et al., 2013; Susan Sundu & Ellen, 2017; Suwedi-Kapesa & Nyondo-Mipando, 2018; Vermeiden, 2019)	Medical care in adjacent/referral health facility perceived as unsatisfactory	
(Abdulkadir, 2017; Bergen et al., 2019; Chibuye et al., 2018; Jarquín & Marenco, 2015; Lori, Wadsworth, et al., 2013; Ruiz et al., 2013; Schooley et al., 2009; Sialubanje et al., 2016; Suwedi-Kapesa & Nyondo-Mipando, 2018; Tiruneh et al., 2016; Vermeiden, Schiffer, et al., 2018)	Medical care in MWH perceived as satisfactory<br>*"Women's decisions to seek care were influenced by the perception that women would be able to access culturally appropriate, safe and secure health care services at the Casa Materna." (Schooley et al., 2009)*	
(Vermeiden, Schiffer, et al., 2018)	Medical care in adjacent/referral health facility perceived as satisfactory	
(Clensay, 2007; Shrestha et al., 2007; Sialubanje et al., 2015; Susan Sundu & Ellen, 2017; Suwedi-Kapesa & Nyondo-Mipando, 2018; Wilson et al., 1997)	Insufficient check-ups by health workers during MWH stay<br>*"The women were not comfortable with the fact that there was no doctor or nurse available on the MWH premises." (Wilson et al., 1997)*	
(Scott et al., 2018; Suwedi-Kapesa & Nyondo-Mipando, 2018)	Lack of medical supplies/equipment<br>*"Health care workers in all the study sites reported that, most of the time, they are not supplied with all the necessary materials needed for the MWHs, for example iron supplements." (Suwedi-Kapesa et al., 2018)*	
(Chibuye et al., 2018; Clensay, 2007; Scott et al., 2018; Suwedi-Kapesa & Nyondo-Mipando, 2018)	Shortage of trained staff	
(García Prado & Cortez, 2012; Kaiser et al., 2019)	Providing postnatal care<br>*"Intervention site staff reported some women waiting up to six days at the MWH in order to attend their six-day postnatal care visit at the health center, rather than travelling the long distances to and from their homes."* (Kaiser et al., 2019)	
(Bergen et al., 2019; Ruiz et al., 2013; Tiruneh et al., 2016)	Health education is unsatisfactory	
(Clensay, 2007; Friedman, 2008a; Jarquín & Marenco, 2015; Lori et al., 2018; van Rijn, 2013; Wester et al., 2018)	Health education is a good addition to MWH's services	
(Bergen et al., 2019; Jarquín & Marenco, 2015; Kebede & Mihrete, 2020; Schooley et al., 2009; Tiruneh et al., 2016; Vermeiden, 2019)	Respectful care by the health workers at MWH	Treatment by health workers
(Chibuye et al., 2018; Clensay, 2007; Kebede & Mihrete, 2020; Lori et al., 2016; Lori, Wadsworth, et al., 2013; Mramba et al., 2010a; Solidar Med, 2013; Susan Sundu & Ellen, 2017; Suwedi-Kapesa & Nyondo-Mipando, 2018; Urwin, 2017; Vermeiden, 2019)	Disrespectful care by health workers at MWH/health facility, no relationship with health workers<br>*"Many raised the concerns about the quality of care they might expect in an MWH; they viewed health facilities as often providing unprofessional and unkind services to women, and worried this might be true for MWHs. […] Participants believe the abuse is a reason women do not go to the facility and suggested it could be a reason to avoid a MWH, even when one is available." (Lori et al., 2016)*	
(Chibuye et al., 2018; García Prado & Cortez, 2012; Kebede & Mihrete, 2020; Solidar Med, 2013)	MWH is perceived as culturally inappropriate (no traditional practices, intergenerational mixing of women)<br>*"Additionally, FGD and KII respondents elaborated on cultural issues raised by the FL respondents. First, respondents believed it was culturally inappropriate to house pregnant women with patient families, travelers or even recently delivered women." (Scott et al., 2018)*	Culturally-appropriate care and traditional practices
(Bergen et al., 2019; Chibuye et al., 2018; García Prado & Cortez, 2012; Ruiz et al., 2013)	Women do not like medical care provided by male doctors	
(Friedman, 2008a; Kebede & Mihrete, 2020; Ruiz et al., 2013; Schooley et al., 2009)	MWH is perceived as culturally appropriate, traditional birth practices are allowed	
(Lori et al., 2020; Lori, Munro, et al., 2013)	Collaboration with trained TBAs	

Third order theme: Economic and geographical accessibility

References	First order themes	Second order themes
(Bergen et al., 2019; Chibuye et al., 2018; Gaym et al., 2012; Kebede & Mihrete, 2020; Lori et al., 2016; Scott et al., 2018; Sialubanje et al., 2016; van Rijn, 2013; Vermeiden, 2019; Wilson et al., 1997)	Hospital fees or additional medical costs are too high<br>*"The main barrier that affected the decision to use MWHs were the lack of funds for food, cleaning materials, and clothes for the mother and neonate needed during and after labor." (Sialubanje et al., 2016)*	Cost for MWH stay
(Clensay, 2007; Gaym et al., 2012; Shrestha et al., 2007; Vermeiden, 2019)	Absence of MWH fees makes MWH use easier	
(Bergen et al., 2019; Clensay, 2007; Jarquín & Marenco, 2015; Kebede & Mihrete, 2020; Kyokan et al., 2016; Lori, Wadsworth, et al., 2013; Sialubanje et al., 2016; van Rijn, 2013; Vian et al., 2017; Wilson et al., 1997)	Living at the MWH is too costly	
(Chibuye et al., 2018; Scott et al., 2018; Sialubanje et al., 2016)	Refusal/shame when not meeting the necessary requirements for delivery<br>*"Nonetheless, some women still gave birth at home for fear of being shamed or criticised by health workers if they had no husbands or husbands who failed to provide supplies, such as baby layettes or bleach, which the facility was often lacking." (Chibuye et al., 2018)*	
(Chibuye et al., 2018; Kyokan et al., 2016; Sitefane et al., 2013)	Fine for non-health facility birth	
(Chibuye et al., 2018; Lori et al., 2016; Vian et al., 2017)	Women would be willing to pay for MWH	
(Bergen et al., 2019; Jarquín & Marenco, 2015; Lori, Wadsworth, et al., 2013; Solidar Med, 2013; Tiruneh et al., 2016; Wilson et al., 1997)	Work and income become affected during absence<br>*"In addition, women said they could not care for other family members and their farms while at the MWHs. This is a problem, because their farms are their main source of livelihood." (Wilson et al., 1997)*	Work and income
(Bergen et al., 2019; Friedman, 2008a; Lori, Wadsworth, et al., 2013; Sitefane et al., 2013)	Cost of transport to MWH is too high<br>*"Costs of transport to referral hospitals from the PHUs can be prohibitive to using services." (Kyokan et al., 2016)*	Transport and distance from household to MWH
(Gaym et al., 2012; Kebede & Mihrete, 2020; Kurji et al., 2019; Kyokan et al., 2016; Lori et al., 2016; Scott et al., 2018; Shrestha et al., 2007; Sitefane et al., 2013; Tiruneh et al., 2016; Vermeiden, 2019)	Transport/distance barriers from household to MWH, e.g. ambulances/vehicles unavailable<br>*"Finally, distance to health care facilities and transportation were topics raised by a majority of participants. Only three women of reproductive age out of 168 (2%) reported access to a car, 24% an oxcart, and 79% a bicycle. In addition, women from the Petauke district stated they are unable to walk at night due to 'fear of wild animals.' Many women noted they must travel exceptionally long distances."* (Lori et al., 2016)	
(Bonawitz et al., 2019; Chibuye et al., 2018; Jarquín & Marenco, 2015; Kaiser et al., 2019; Kyokan et al., 2016; Lori, Wadsworth, et al., 2013; Sialubanje et al., 2016; Susan Sundu & Ellen, 2017; Suwedi-Kapesa & Nyondo-Mipando, 2018; Urwin, 2017; van Rijn, 2013; Vermeiden, Schiffer, et al., 2018)	MWH is lowering the geographical barrier to access healthcare	
(Bergen et al., 2019; Chibuye et al., 2018; van Rijn, 2013)	Support of family depends on distance household to MWH	
(Jarquín & Marenco, 2015; Kyokan et al., 2016)	Cost of transport to referral hospital is too high	Transport and distance MWH to adjacent or referral health facility
(Abdulkadir, 2017; Bergen et al., 2019; Chibuye et al., 2018; Friedman, 2008a; Kyokan et al., 2016; Sialubanje et al., 2016; Suwedi-Kapesa & Nyondo-Mipando, 2018; Vermeiden, Schiffer, et al., 2018; Wilson et al., 1997)	Transport or distance challenges MWH to adjacent/referral health facility	

Third order theme: Maternity Waiting Home's characteristics

References	First order themes	Second order themes
(Bonawitz et al., 2019; Lori et al., 2016; Lori, Wadsworth, et al., 2013; Ruiz et al., 2013; Shrestha et al., 2007; Sialubanje et al., 2016; Tiruneh et al., 2016)	Facility for companions is available / companions at MWH are allowed<br>"FGDs respondents discussed the importance of having a companion with them at the MWH: "If you have someone to look after you here it becomes easy to use the MWH." – Waiting woman, Nyimba" (Bonawitz et al., 2019)	Companions
(Gaym et al., 2012; Suwedi-Kapesa & Nyondo-Mipando, 2018; Vermeiden, Schiffer, et al., 2018)	No facility available for companion / companions not allowed	
(Chibuye et al., 2018; Tiruneh et al., 2016; Vermeiden, Schiffer, et al., 2018)	Infrastructure/state/hygiene is satisfactory	Infrastructure and capacity
(Clensay, 2007; Sialubanje et al., 2016; Sitefane et al., 2013; Susan Sundu & Ellen, 2017)	Infrastructure/state/hygiene is unsatisfactory<br>*"A further barrier affecting the use of MWHs was their poor and deplorable state."(Sialubanje et al., 2016)*	
(Bergen et al., 2019; Bonawitz et al., 2019; Chibuye et al., 2018; Lori et al., 2020; Scott et al., 2018; Sialubanje et al., 2015, 2016; Susan Sundu & Ellen, 2017; Suwedi-Kapesa & Nyondo-Mipando, 2018; Tiruneh et al., 2016; van Rijn, 2013; Vermeiden, 2019; Vermeiden, Schiffer, et al., 2018)	Capacity is insufficient, MWH is overcrowded	
(Chibuye et al., 2018; Jarquín & Marenco, 2015; Lori et al., 2017; Sialubanje et al., 2015; Suwedi-Kapesa & Nyondo-Mipando, 2018; Tiruneh et al., 2016; Urwin, 2017)	Basic facilities and services are satisfactory<br>*"Women were generally satisfied with the quality of the newly-constructed MWHs and appreciated having available amenities including beds, mattresses, blankets, mosquito nets, and cooking utensils." (Bonawitz et al., 2019)*	Provision of basic services and facilities
(Friedman, 2008a; Shrestha et al., 2007)	Basic facilities are unsatisfactory	
(Bergen et al., 2019; Bonawitz et al., 2019; Chibuye et al., 2018; Scott et al., 2018; Sialubanje et al., 2016; Susan Sundu & Ellen, 2017; Suwedi-Kapesa & Nyondo-Mipando, 2018)	Lack of latrines/sanitary conditions	
(Chibuye et al., 2018; Kaiser et al., 2019; Lori et al., 2017; Suwedi-Kapesa & Nyondo-Mipando, 2018; Vermeiden, Schiffer, et al., 2018)	Lack of electricity	
(Bergen et al., 2019; Bonawitz et al., 2019; Chibuye et al., 2018; Lori et al., 2020; Scott et al., 2018; Sialubanje et al., 2016; Tiruneh et al., 2016; Vermeiden, Schiffer, et al., 2018)	Lack of beds and clean bedding<br>*"Comfort, particularly around overcrowding, was the primary theme elicited in the FL responses and corroborated by FGD and KII respondents. They explained that the MWHs were crowded, had no beds or mattresses, limited access to water, and were generally uncomfortable." (Scott et al., 2018)*	
(Chibuye et al., 2018; Lori et al., 2017)	Daily activities are satisfactory	
(Chibuye et al., 2018; Clensay, 2007; Suwedi-Kapesa & Nyondo-Mipando, 2018; Tiruneh et al., 2016; van Rijn, 2013)	Lack of entertainment, feeling of boredom	
(Bergen et al., 2019; Bonawitz et al., 2019; Shrestha et al., 2007; Suwedi-Kapesa & Nyondo-Mipando, 2018; Tiruneh et al., 2016)	Lack of cooking facilities, cooking utensils unavailable<br>*"Similar to the quantitative findings, women identified inadequate storage space for their food. Even after implementation at the intervention sites, women complained that the cooking area was too small and dirty." (Bonawitz et al., 2019)*	
(Mramba et al., 2010b; Shrestha et al., 2007; Suwedi-Kapesa & Nyondo-Mipando, 2018)	Availability of mosquito nets	
(Bergen et al., 2019; Chibuye et al., 2018; James et al., 2019; Jarquín & Marenco, 2015; Kyokan et al., 2016; Mramba et al., 2010a; Shrestha et al., 2007; Sialubanje et al., 2015; Wester et al., 2018)	Food is provided and satisfactory	Provision of food
(Chibuye et al., 2018; Kaiser et al., 2019; Kebede & Mihrete, 2020; Lori et al., 2017; Scott et al., 2018; Sialubanje et al., 2015; Vermeiden, Schiffer, et al., 2018)	Limited access to clean drinking water<br>*"Participants at Mulanje District Hospital MWH reported that they do not always have a water supply within the facility and would draw water from a river. Although the buildings have indoor plumbing, the water supply is inconsistent even if the water bill is paid." (Suwedi-Kapesa et al., 2018)*	
(Bergen et al., 2019; Chibuye et al., 2018; Clensay, 2007; Gaym et al., 2012; Kebede & Mihrete, 2020;	Food insecurity, food is unsatisfactory	

Kyokan et al., 2016; Lori et al., 2020; Lori, Wadsworth, et al., 2013; Pujihartati et al., 2019; Sitefane et al., 2013; Solidar Med, 2013; Suwedi-Kapesa & Nyondo-Mipando, 2018; Tiruneh et al., 2016; van Rijn, 2013; Vian et al., 2017)	*"Key informants reported that non provision of food at the BWHs is a barrier to women's use of BWH. Some non-users reported that they expected the BWHs to provide food, whereas others understood that they should supply their own food." (Kyokan et al., 2016)*	
(Chibuye et al., 2018; Gaym et al., 2012; Lori, Wadsworth, et al., 2013; Tiruneh et al., 2019; van Rijn, 2013)	Access to food depends on family support	
(Chibuye et al., 2018; Scott et al., 2018)	Security and safety should be provided<br>*"A CRHC maternity home user said she preferred giving birth at one of the mission hospitals where there was a fence, for security reasons. She suggested having a watchman at night for protection." (Chibuye et al., 2018)*	Security and safety
(Scott et al., 2018)	No lockable space for the belongings, women steal from each other	

Third order theme: Organizational structures and community engagement

References	First order themes	Second order themes
(Abdulkadir, 2017; Bergen et al., 2019; Chibuye et al., 2018; García Prado & Cortez, 2012; Kaiser et al., 2019; Kebede & Mihrete, 2020; Lori et al., 2020; Lori, Wadsworth, et al., 2013; Scott et al., 2018; Tiruneh et al., 2016)	Active participation and ownership of the community<br>*"The CMWHs should be constructed by the community using local materials to achieve optimal community ownership." (Wester et al., 2018)*	Community engagement
(Bergen et al., 2019; Kebede & Mihrete, 2020; Schooley et al., 2009; Scott et al., 2018)	Communities contribute financially	
(García Prado & Cortez, 2012; Gaym et al., 2012; Scott et al., 2018; Tiruneh et al., 2016)	Need for standardizing management process improvement, MWH guidelines	Management and responsibility
(Abdulkadir, 2017; Chibuye et al., 2018; Scott et al., 2018)	Shortage and high workload of medical staff<br>*"HCWs felt overworked, underpaid and undertrained." (Vermeiden et al.2019)*	
(Abdulkadir, 2017; Chibuye et al., 2018; García Prado & Cortez, 2012; Gaym et al., 2012; Kebede & Mihrete, 2020; Ruiz et al., 2013; Tiruneh et al., 2016)	Lack of funding, financial deficits<br>*"Several participants explained that the home had always been financed by the municipality. This changed with the election of the new local mayor who did not give priority to the MWH and resources stopped. All Cuilco participants perceived the lack of sustainable funding as a serious problem." (Ruiz et al., 2013)*	
(Bergen et al., 2019; Chibuye et al., 2018; Clensay, 2007; Friedman, 2008b; García Prado & Cortez, 2012; Kebede & Mihrete, 2020; Schooley et al., 2009; Scott et al., 2018; van Rijn, 2013)	More responsibility for the national government	
(Bergen et al., 2019; Chibuye et al., 2018; Kyokan et al., 2016; Lori et al., 2020; Schooley et al., 2009; Vermeiden, Braat, et al., 2018; Wilson et al., 1997)	Advocacy and referral by TBAs/community leaders/HEWs	Referral and advocacy of MWHs
(Kyokan et al., 2016)	TBAs are not willing to refer women due to loss of income	
(Chibuye et al., 2018; Pujihartati et al., 2020; Schooley et al., 2009; Sialubanje et al., 2015; Vermeiden, 2019; Wester et al., 2018)	"Recommendation and referral by MWHs by health workers<br>Another successful practice is for health workers themselves to promote the use of maternity homes at their CRHC and build community support." (Chibuye et al., 2018)	
(Bergen et al., 2019; Clensay, 2007; Kebede & Mihrete, 2020; Schooley et al., 2009; van Rijn, 2013)	Word-to-mouth is an effective strategy for promoting MWHs<br>*"After good outcomes, the news is promoted by users in their villages and eventually from one generation to the next. Place of residence of MWH users revealed that positive word-of-mouth spread far beyond the boundaries of the PH programme." (Vermeiden et al., 2018)*	
(Friedman, 2008b; Gaym et al., 2012; Jarquín & Marenco, 2015; Kyokan et al., 2016; Ruiz et al., 2013)	Strong referral system and partnership between various levels (e.g. MWH and referral health facility)	
(Bergen et al., 2019; García Prado & Cortez, 2012)	Experiences of former users	
