# Supplementary material for: Stakeholders’ perspectives on the acceptability and feasibility of maternity waiting homes: a qualitative synthesis
Source: Reprod Health. 2023 Jul 5;20:101. doi: 10.1186/s12978-023-01615-x (PMC10324180; doi:10.1186/s12978-023-01615-x)
Supplement: Supplementary file 5 — Additional file 5: Appendix S4. Perceived gains/facilitators and perceived losses/barriers. [file 12978_2023_1615_MOESM5_ESM.pdf]

***Additional file 5.*** *Appendix S4 Perceived gains/facilitators and perceived losses/barriers*

| | **Perceived gains/facilitators** | **Perceived losses/barriers** |
|---|---|---|
| **Individual factors** | Better health outcomes | No perceived benefits |
| | Negative experience with home-birth | Positive experience with home-birth |
| | High awareness of existence MWH | Low awareness of existence MWH |
| | Better monitoring by health workers | Individual preference for natural birth |
| | Health education | Fear of operation |
| | Free lodging | Negative experience facility-birth or other aspects of the health system |
| | Easier access to health care | Uncertainty about how and when to use MWH |
| | Sharing experiences with other women | |
| | Easier to plan work, earlier recognition of complications | |
| | Resting place, being away from domestic responsibilities | |
| **Interpersonal factors and family commitments** | Male partner supports MWH use | Male partner prohibits MWH use |
| | Mother-in-law or other family members support MWH use | Lack of support from family members |
| | Support from family members | Domestic responsibilities, no one to take over the care for other children/household |
| | Others can take over the care for children/household | Fear of adultery during absence |
| | Community is positive about MWH, support women to go | Community is negative about MWH or women that use them |
| **Financial and geographical accessibility** | Fine for home-birth | Additional cost for birth in adjacent health facility is too high |
| | Willingness to pay for MWH | Cost of living at MWH is too high |
| | | Work and income become negatively affected |
| | | Transportation difficulties household to MWH |
| | | Transportation difficulties MWH to adjacent health facility |
| | | MWH too far from household |
| **MWH's characteristics** | Basic facilities are satisfactory: availability of electricity, | Basic facilities are unsatisfactory: lack of electricity, sanitary facilities, cooking facilities and mosquito nets |

| | | |
|---|---|---|
| | sanitary facilities, cooking utensils | |
| | Feeling secure and safe | Poor state and low capacity |
| | Daily activities available | Lack of programming |
| | Facility for companions | No option for bringing companions |
| | Food and clean water is provided and satisfactory | Food and clean water insecurity |
| **QOC** | Quality of MWH is satisfactory | Quality of MWH is unsatisfactory |
| | Quality of care in adjacent or referral health facility is satisfactory | Quality of care in adjacent or referral health facility is unsatisfactory |
| | Respectful care by health workers | Disrespectful treatment by health workers |
| | Integration of cultural aspects, e.g. traditional birth practices or assistance by TBA | Culturally inappropriate care, e.g. male health workers, prohibition traditional birth practices |
| **Organization and advocacy** | Advocacy by TBAs, community leaders, community workers, SMAGs in community | TBA not willing to refer women to MWH |
| | Advocacy by health workers in facilities and ANC clinics | Financial deficits, funding not sustainable |
| | Word-of-mouth promotion of former users | Lack of standardized guidelines |
| | Increased governmental support | Lack of governmental support |
| | Incorporation of trained TBAs | Increased workload for health workers |
| | Active involvement of the community | |
| | Financial contribution of community | |
